# Bioactive Marine Xanthones: A Review

**DOI:** 10.3390/md20010058

**Published:** 2022-01-08

**Authors:** José X. Soares, Daniela R. P. Loureiro, Ana Laura Dias, Salete Reis, Madalena M. M. Pinto, Carlos M. M. Afonso

**Affiliations:** 1 Laboratório Associado para a Química Verde (LAQV), Rede de Química e Tecnologia (REQUIMTE), Department of Chemical Sciences, Faculty of Pharmacy, University of Porto, Rua de Jorge Viterbo Ferreira, 228, 4050-313 Porto, Portugal; jfxsoares@ff.up.pt (J.X.S.); dloureiro@ff.up.pt (D.R.P.L.); shreis@ff.up.pt (S.R.); 2 Laboratory of Organic and Pharmaceutical Chemistry, Department of Chemical Sciences, Faculty of Pharmacy, University of Porto, Rua de Jorge Viterbo Ferreira, 228, 4050-313 Porto, Portugal; up201903848@ff.up.pt (A.L.D.); madalena@ff.up.pt (M.M.M.P.); 3 Interdisciplinary Center of Marine and Environmental Investigation (CIIMAR/CIMAR), Edifício do Terminal de Cruzeiros do Porto de Leixões, Av. General Norton de Matos s/n, 4050-208 Matosinhos, Portugal

**Keywords:** xanthone, marine products, natural products, molecular descriptors, antimicrobial, antitumor, drug-like

## Abstract

The marine environment is an important source of specialized metabolites with valuable biological activities. Xanthones are a relevant chemical class of specialized metabolites found in this environment due to their structural variety and their biological activities. In this work, a comprehensive literature review of marine xanthones reported up to now was performed. A large number of bioactive xanthone derivatives (169) were identified, and their structures, biological activities, and natural sources were described. To characterize the chemical space occupied by marine-derived xanthones, molecular descriptors were calculated. For the analysis of the molecular descriptors, the xanthone derivatives were grouped into five structural categories (simple, prenylated, *O*-heterocyclic, complex, and hydroxanthones) and six biological activities (antitumor, antibacterial, antidiabetic, antifungal, antiviral, and miscellaneous). Moreover, the natural product-likeness and the drug-likeness of marine xanthones were also assessed. Marine xanthone derivatives are rewarding bioactive compounds and constitute a promising starting point for the design of other novel bioactive molecules.

## 1. Introduction

The marine environment occupies more than half of the Earth’s surface and harbors the largest pool of biodiversity. Among others, marine microorganisms produce specialized metabolites used mostly in interspecies competition and defense from predators. The harsh conditions found in the sea spurs the development of specific biosynthetic pathways that produce metabolites bearing novel scaffolds, quite different from those found in terrestrial sources [1]. Specialized metabolites were optimized by evolution to establish flawless interactions with biological targets [2]. The sum of these factors makes the marine environment a prolific source of structurally diverse bioactive molecules that have a pharmacological interest [3].

Among the most relevant chemical classes of specialized metabolites isolated from the marine biodiversity, xanthones are a class of oxygen-heterocycles containing a heterocycle containing a dibenzo γ-pyrone moiety [4,5,6]. Depending on the nature and position of substituents, xanthone derivatives show a wide variety of biological activities making the xanthone scaffold a “privileged structure” in Medicinal Chemistry with a good potential for the discovery of novel hits, leads, and drugs [7].

The chemical space occupied by a collection of substances is usually mapped by molecular descriptors [8]. Molecular descriptors are generically defined as mathematical representations of molecular features and embrace a vast collection of molecular, physicochemical, and topological parameters. Each molecular feature is encoded by at least one molecular descriptor. Size is usually inferred by the molecular weight (MW); flexibility by the number of rotatable bonds; lipophilicity by partition coefficient between octanol and water (Log P); polarity by the topological polar surface area (TPSA); solubility by the logarithm of the solubility measured in mol L^−1^ (log S); and carbon saturation by the fraction of sp^3^ carbons (Fsp^3^). Besides providing a numerical expression for chemical features, molecular descriptors allow tracking the suitable pharmacodynamics and pharmacokinetics properties, i.e., allow pursuing drug-likeness. Sets of rules or filters have been proposed over time in order to predict pharmacokinetic behavior. The most common set is the Lipinski′s rule of five [9], but other approaches have been suggested by other authors, namely by Veber [10], Ghoose [11], Egan [12], and Gleeson [13]. More recently, Bickerton et al. proposed the quantitative estimate of drug-likeness (QED) based on the calculation of the desirability of eight molecular properties [14]. Due to its usefulness in mapping the drug-likeness territory, this model has also been expanded to natural products (NP), creating the concept of NP-likeness evaluated by a score that allows comparing the chemical space covered by NPs with the one covered by synthetic molecules (SM) [15]. The application of this score helps medicinal chemists design molecules that are inspired by nature and have a higher probability of having a suitable pharmacokinetic behavior [16].

In this work, we review 169 bioactive marine xanthone derivatives and present their structures, biological activities, and marine sources. The chemical space occupied by bioactive marine xanthones is mapped and framed according to the NP-likeness and drug-likeness concepts.

## 2. Bioactive Xanthones Isolated from the Marine Environment

The bibliographic research was conducted using Scopus^®^, Web of Science^®^, and Google Scholar^®^ without any temporal restriction. The keywords used were “marine AND xanthone*”.

**Figure 1:**
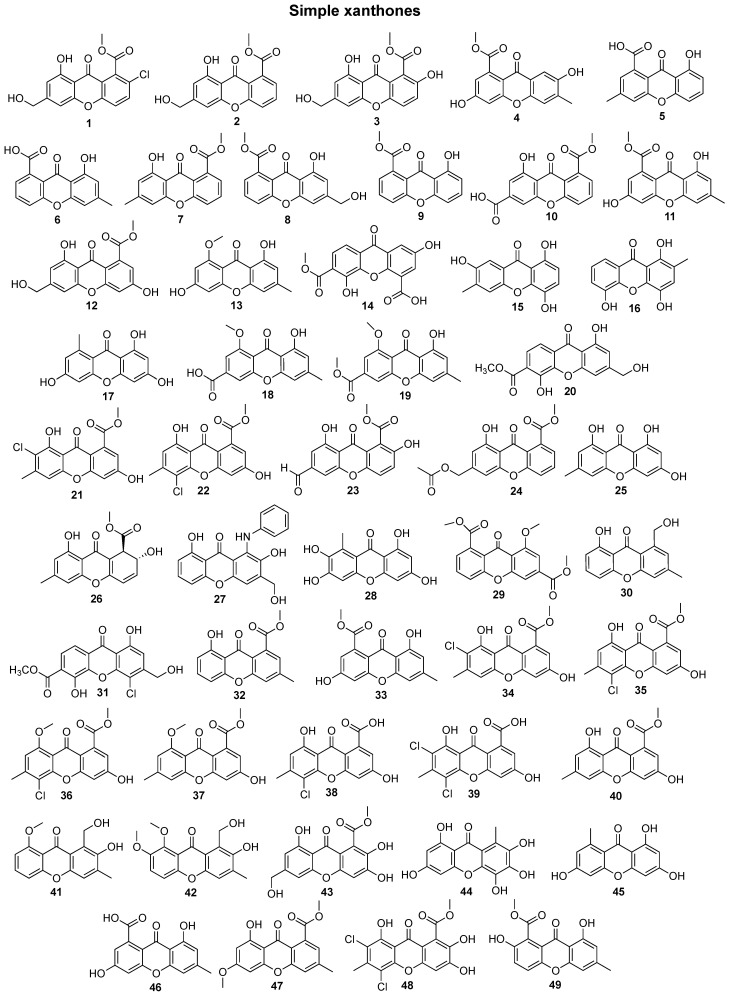

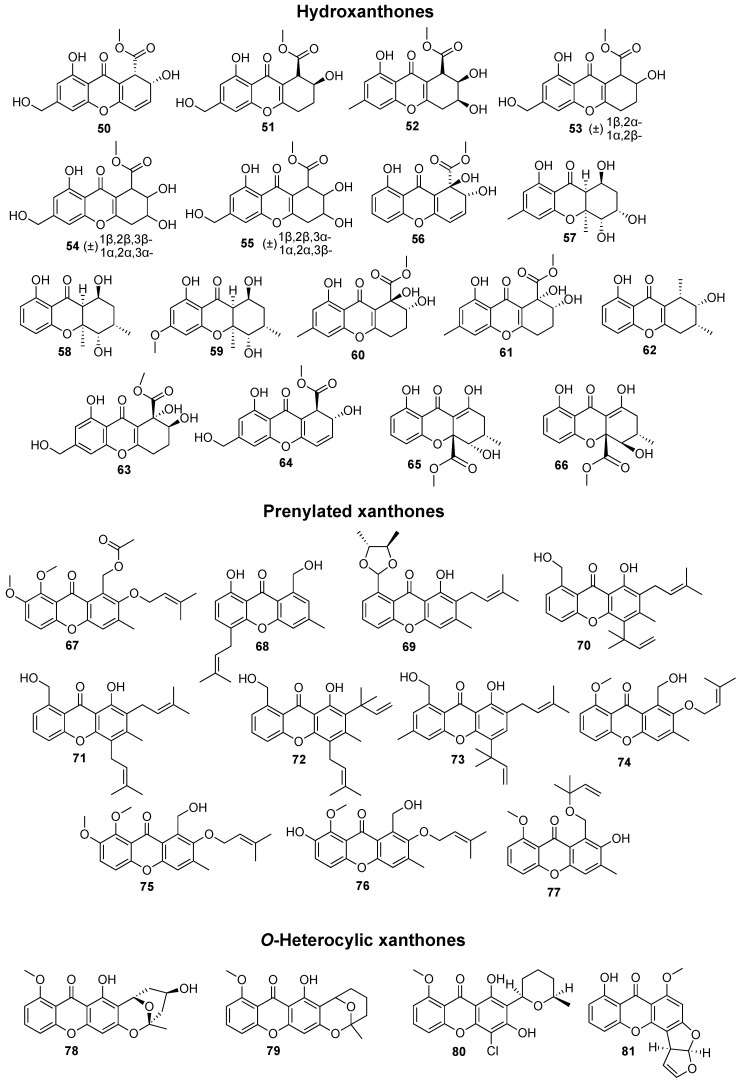

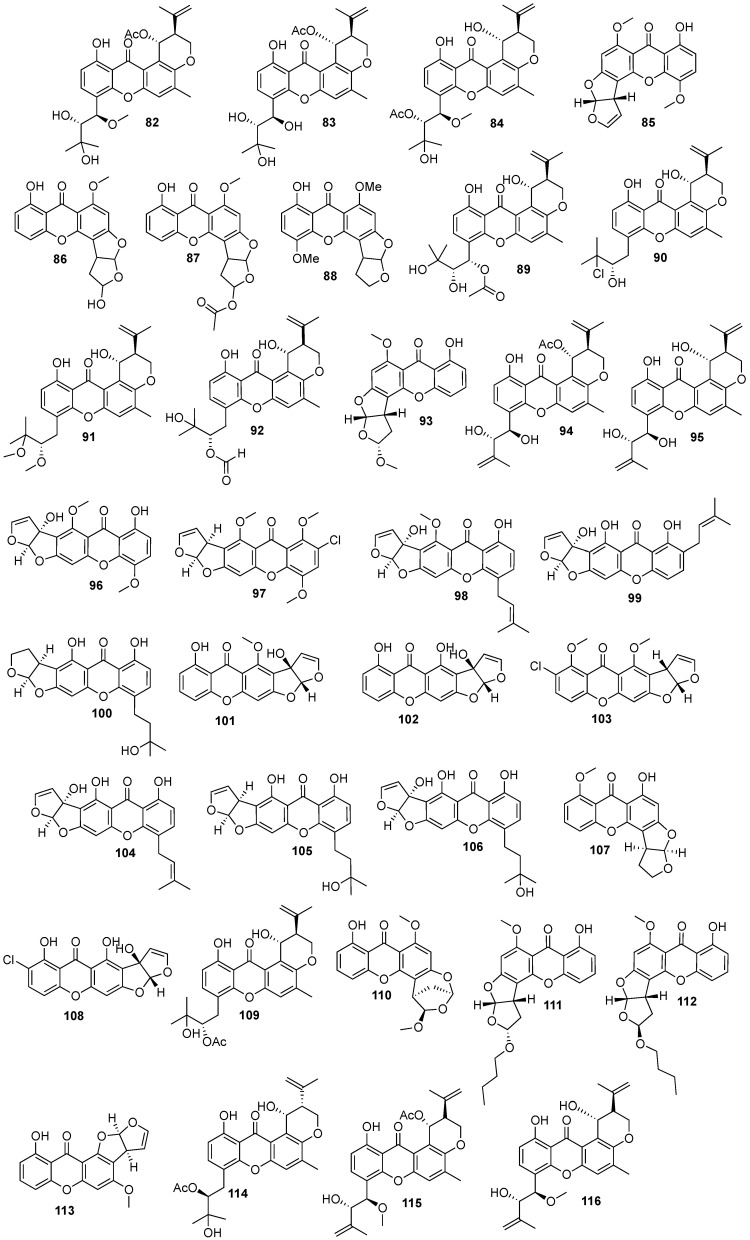

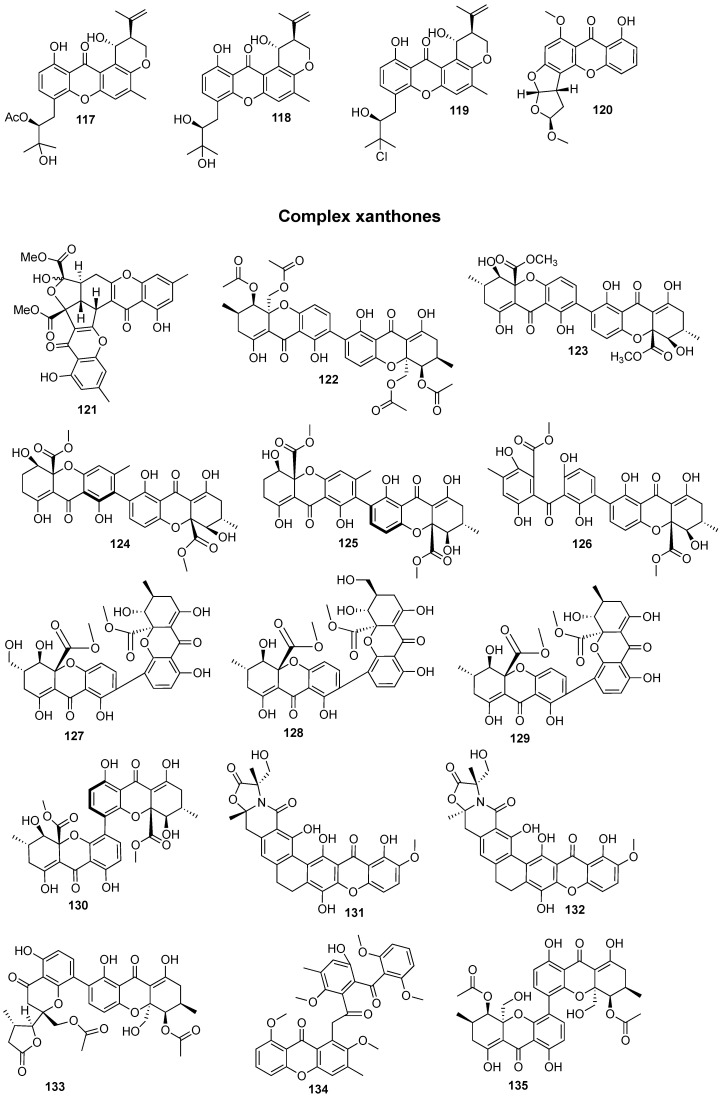

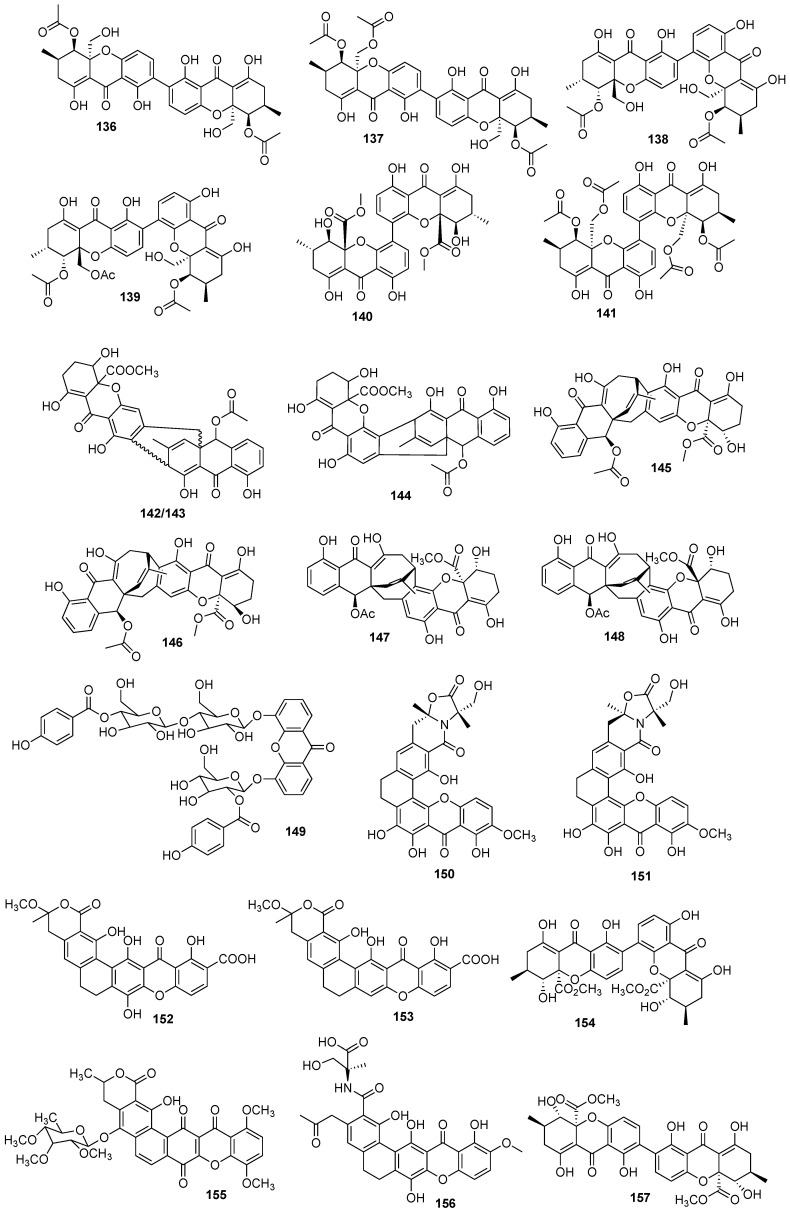

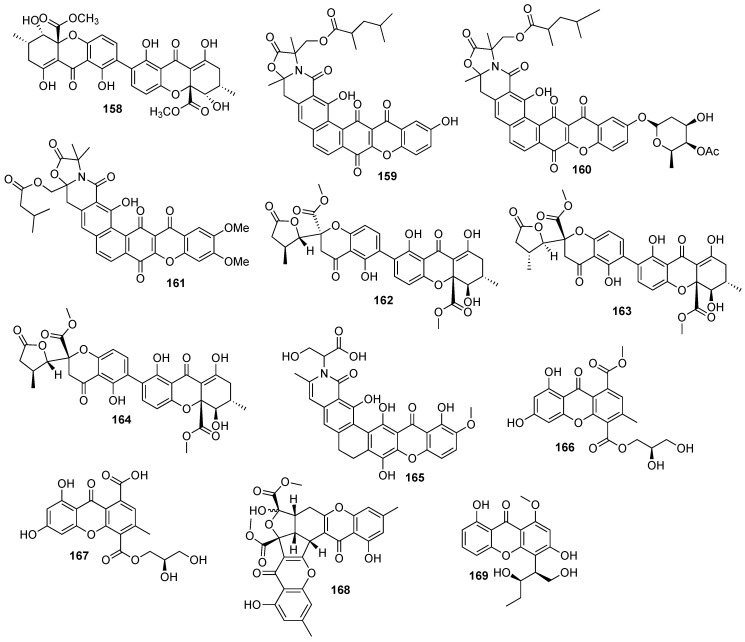
Structures of bioactive marine xanthones.

In total, 169 xanthones derivatives were identified, which were sorted into 5 different structural categories (Figure 1). Simple oxygenated xanthones, bearing substituents such as hydroxyl, carboxyl, and methoxy groups, were classified as “simple” xanthones. Xanthones bearing isoprenyl groups were classified as “prenylated” compounds. Xanthones bearing additional *O*-heterocyclic groups, such a pyran or furan ring, were classified as “*O*-heterocyclic” compounds. Xanthones bearing *O*-heterocyclic and isoprenyl groups were classified as “*O*-heterocyclic” because they have a higher similarity with this category. Dimeric, pseudo-dimeric (one xanthonic and a hydroxanthone nucleus connected by a C-C bond), and glycosylated xanthones were loosely classified as “complex”. Dihydro-, tetrahydro-, and hexahydroxanthones were included in the “hydroxanthone” category (Figure 1).

**Figure 2:**
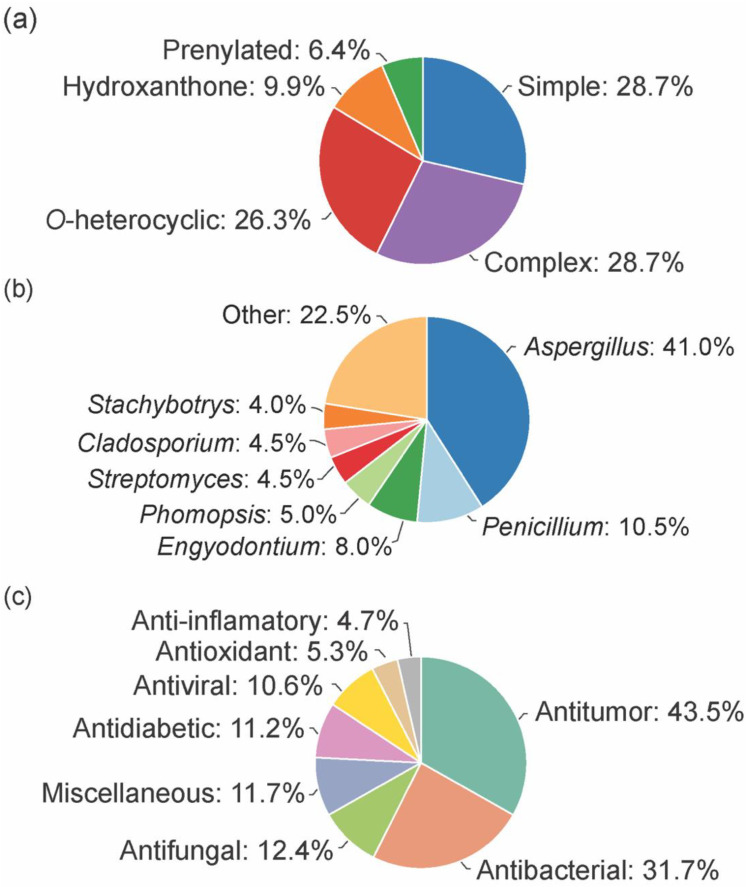
Distribution of type (**a**) microorganism source (**b**) and activity (**c**) of the bioactive marine xanthones.

From the considered 5 different structural types, “simple” (28.7%) and “complex” (28.7%) groups were the most prevalent, followed by “*O*-heterocyclic” (26.3%), “hydroxanthones” (9.9%), and “prenylated” (6.4%) (Figure 2a).

The bioactive marine xanthones were mostly isolated from marine fungi, namely from fungi belonging to the *Aspergillus* genus (41%, Figure 2b). Only a few examples were isolated from bacteria, and among them, the *Streptomyces* genus was the source that provided more bioactive xanthones (4.5%). Half of the reported bioactive marine xanthones (53%) were isolated from endophytic microorganisms associated with macroorganisms, like mangroves (39 marine xanthones), sponges (38 marine xanthones), algae (16 marine xanthones), corals (12 marine xanthones), jellyfish (1 marine xanthone), and seaweed (1 marine xanthone).

As specialized metabolites, xanthones are often used as chemical defense agents. The most prevalent described activities were antitumor (43.5%) and antimicrobial (antibacterial (31.7%), antifungal (12.4%), and antiviral (10.6%) which provides some sort of protection to other competitive or predator marine organisms (Figure 2c). Interestingly, 22% of the identified marine xanthones presented more than one biological activity (sum of all activities >100% in Figure 2c).

## 3. Chemical Space of Bioactive Marine Xanthones

Bioactive marine xanthones are produced by and act in living organisms, and to fulfill their specific biological task, they are structurally optimized by nature. Therefore, defining their chemical space is important for designing new molecules with desirable properties and pharmacological potential.

**Figure 3:**
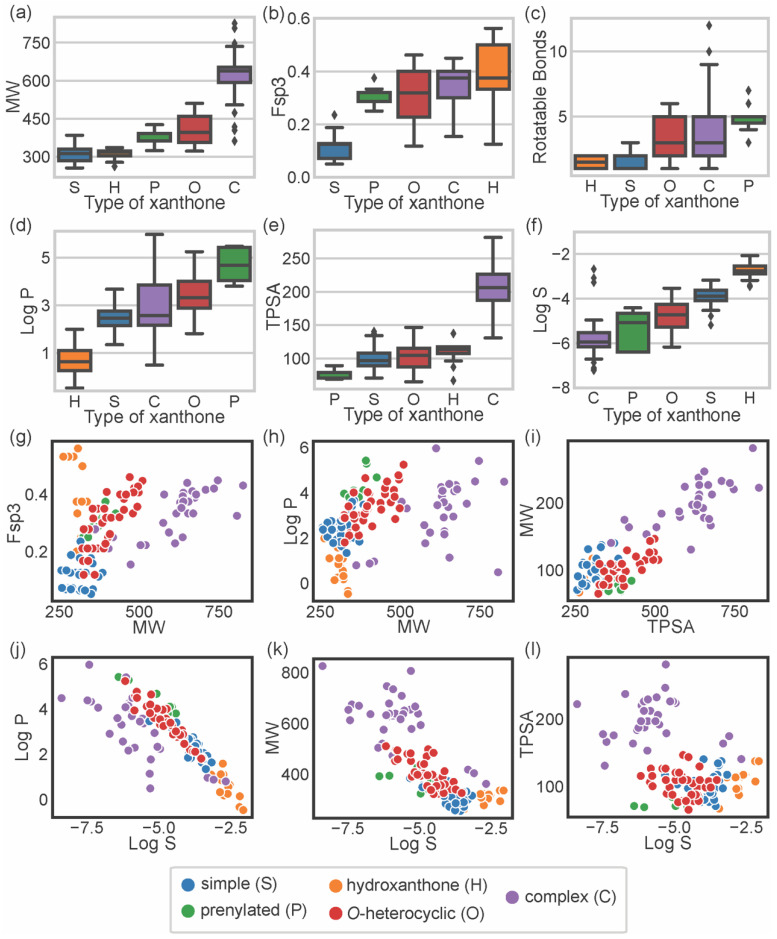
Distribution of MW (**a**); Fsp^3^ (**b**); number of RBs (**c**); log P (**d**); TPSA (**e**); and log S (**f**) accordingly to the type of xanthone: “simple” (S, blue), “prenylated” (P, green), “hydroxanthone” (H, orange), *O*-heterocyclic (O, red), and complex (C, purple). Comparison between the values of Fsp^3^ carbons and MW (**g**); log P and MW (**h**); MW and TPSA (**i**); log P and log S (**j**); MW and log S (**k**); TPSA and log S (**l**).

To describe the chemical space occupied by bioactive marine xanthones, several molecular descriptors, embracing different molecular, physico-chemical, and topological properties, were calculated using the RDkit (release 2021_03_5 Q1 2021) and SwissADME [17]. For each marine xanthone, the following molecular descriptors were calculated: molecular weight (MW), fraction of sp3 carbons (Fsp3), number of rotatable bonds (RB), lipophilicity (Log P), topological polar surface area (TPSA), and solubility (Log S) (Table S1). The molecular descriptors were analyzed accordingly to the structural type of xanthones (Figure 3).

Molecular size can be expressed in terms of MW, which is a predictor for pharmacokinetics behavior because bioavailability usually decreases as the molecular size increases [18]. In terms of size, the majority of the marine xanthones presented MWs within the range of 300 to 600 g·mol^−1^. “Complex” xanthones presented the highest mean value (621.7 g·mol^−1^) and the highest value dispersion (standard deviation of 85) due to the inclusion of several dimeric and glycosylated structures. On the other side, “simple” xanthones and “hydroxanthones” have the lowest MW mean values (306.9 and 311.2 g·mol^−1^, respectively) as these xanthones are composed by the xanthonic nucleus with simple substituents (such as hydroxyl or methyl or methoxy group).

Molecular flexibility depends primarily on carbon saturation (Fsp^3^) and on the number of rotatable bonds (RB). A high number of RB and/or Fsp3 means that the molecule has conformation flexibility which results in a less planar, less rigid, and more complex three-dimensional shape. Both RB and Fsp3 are important for determining oral bioavailability [10,19]. “Simple” xanthones have the lowest Fsp^3^ values (mean value of 0.11) as saturated bonds are only present in substituent groups (Figure 3b). “Prenylated” and “*O*-heterocyclic” xanthones have equal mean Fsp^3^ values (mean values of 0.31), but the latter showed higher value dispersion (interquartile range of 0.03 for “prenylated” and 0.17 for “*O*-heterocyclic” xanthones, Figure 3b), which is a consequence of higher 3D complexity rather than larger size (Figure 3g). “Complex” xanthones have a high value dispersion (standard deviation of 0.07, Figure 3b) due to their wide range of sizes (Figure 3g). Fsp^3^ values greater than 0.42 are considered to be suitable values for a drug [19], and half of the “hydroxanthones” obey this criterion. In agreement with Fsp^3^ analysis, “*O*-heterocyclic”, “complex”, and “prenylated” xanthones have a higher number of freely rotating bonds (median values of 3, 3, and 5 for “*O*-heterocyclic”, “complex”, and “prenylated”, respectively). “Hydroxanthones” have the highest Fsp^3^ values (median value of 0.38), but they have the lowest number of RB (median value of 1.50), meaning that saturated bonds belong to the cyclic system of the hydroxanthonic nucleus (Figure 3c). Good oral absorption is associated with a number of RBs < 10 [10], and the vast majority of the marine xanthones fulfill this criterion.

Lipophilicity, assessed by log P, is a key parameter that affects both pharmacodynamics and pharmacokinetics [20]. “Hydroxanthone” (mean value of 0.68) and “simple” xanthones (mean value of 2.48) have the lowest log P values as they are frequently substituted with hydrophilic groups (hydroxyl and carboxylic). “*O*-heterocyclic” xanthones presented log P values similar (median value of 3.32) to the xanthone itself (calculated log P of 2.95), meaning that the additional *O*-heterocyclic moiety does not contribute significantly to lipophilicity. “Prenylated” xanthones have the highest log P value (mean value of 4.68), significantly higher than the “*O*-heterocyclic”. “Complex” xanthones (median of 2.57) present the highest dispersion of log P values (standard deviation of 1.2), putting in evidence their structural diversity. The increase or decrease lipophilicity of marine xanthones is dependent on the substitution pattern, namely on the presence of hydrophilic or lipophilic substitutions. The size of the marine xanthone was not correlated with increasing lipophilicity as different sized molecules have quite similar log P values (Figure 3h), such as compound **10** (314 g·mol^−1^, log P 2.14) and compound **157** (638 g·mol^−1^, log P 2.16).

Molecular polarity, evaluated as the sum of surfaces of polar atoms in a molecule (TPSA), has been used to predict the permeability of drugs [21]. Different xanthone types have quite similar mean TPSA values (ranged from 74.8 Å^2^ for “prenylated” up to 110.7 Å^2^ for “hydroxanthones”), with the exception of complex xanthones that have significantly higher values (mean value of 203.9 Å^2^) (Figure 3e). “Complex” xanthones are the only type that violates the preconized 140 Å^2^ limit value [10]. In marine xanthones, the polarity is mostly related to MW, as TPSA values increase almost linearly with the MW (Figure 3i). In the case of marine xanthones, this is attributed to the increased number of polar atoms, such as oxygen or nitrogen, with increasing MW.

The water solubility, expressed as log S, is an important parameter for drug bioavailability. Compounds with poor water solubility have poor absorption and oral bioavailability, the evaluation of their bioactivity might be erratic, and the formulation development will be challenging [22]. The solubility trend observed with marine xanthones was: “hydroxanthones” > “simple” > “*O*-heterocyclic” > “prenylated” > “complex”. “Hydroxanthones” were the most soluble group (mean value of −2.74), while “complex” xanthones were the most poorly soluble group (mean value of −5.84) (Figure 3f). “Simple” and “hydroxanthones” are above the log S value of −4, which is considered an acceptable value for a drug [23]. This trend is related to lipophilicity (Figure 3j) and size (Figure 3k). The poor solubility of complex xanthones is ascribed to their high molecular weight and high lipophilicity, while the smaller and/or more hydrophilic hydroxanthones have good water solubility (Figure 3f). Solubility of marine xanthones seems not to be affected by polarity as log S and TPSA were not correlated (Figure 3l).

**Figure 4:**
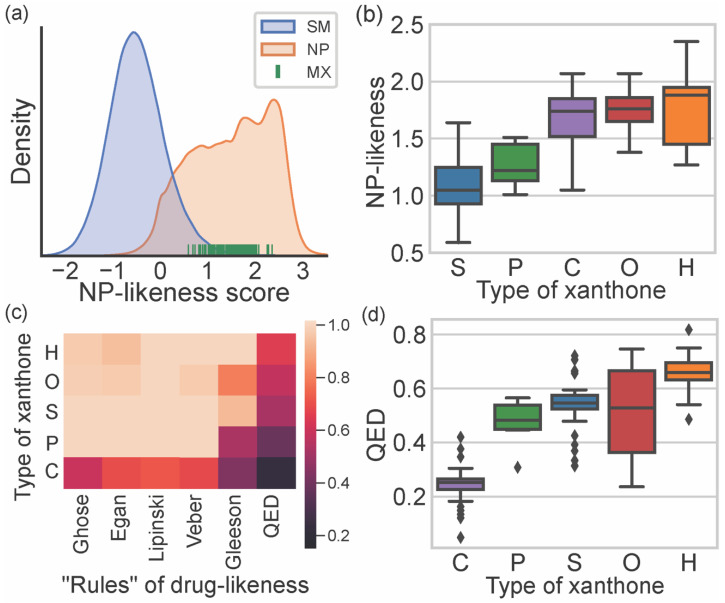
(**a**) KDE distribution plot NP-likeness score of synthetic molecules (SM), natural products (NP), and NP-likeness score of marine xanthones. (**b**) Distribution of NP-likeness score accordingly to the type of xanthone: “simple” (S, blue), “prenylated” (P, green), “hydroxanthone” (H, orange), “*O*-heterocyclic” (O, red), and “complex” (C, purple). (**c**) Heatmap of the compliance with rules of drug-likeness for the xanthone types. (**d**) Distribution of QED index accordingly to the type of xanthone.

NP-likeness allows measuring the similarity of a molecule to natural products [15]. The NP-likeness score quantifies this similarity; the higher the score, the higher the resemblance of that molecule to an NP [15]. NP-likeness scores of marine xanthones were calculated using the web service NaPles [24]. Figure 4a depicts the probability density function, based on kernel density estimation (KDE), of NP-likeness score for all NPs and synthetic molecules (SMs), as well as the score of marine xanthones. As expected, the NP-likeness score of marine xanthones falls within the range of NP. NP-likeness score of marine xanthones was analyzed considering the different types of xanthones (Figure 4b). Xanthone itself is similar to SMs (NP-likeness score of 0.19), while hexahydroxanthone presents a high score (NP-likeness score of 1.57). Within marine xanthones, “simple” xanthones have the lowest similarity with NP molecules (mean score of 1.09). The extension of the degree of substitution, from the simple hydroxyl groups in “simple” xanthones, up to an additional xanthonic nucleus present in “complex” xanthones, leads to the increase in the similarity to NP. This is in agreement with the fact that usually, NP are structurally more complex than synthetic molecules [25].

Drug-likeness allows estimating the probability of a molecule to become a drug administered orally [17]. The classical approach to drug-likeness is normally based on a set of criteria to which the compounds under study should obey. This approach provides a binary “yes or no” assessment, depending on if the compound obeys or not the preconized limit values. Drug-likeness of marine xanthones were evaluated considering the classical Lipinski [9], Veber [10], Ghoose [11], Egan [12], and Gleeson rules [13]. In this study, a compliance value, defined as 0 when a compound does not obey any of the preconized criteria of that rule and 1 when a compound fulfills all criteria, was calculated for each marine xanthones (Table S2). Figure 4c displays the obtained mean compliance values of each type of marine xanthones for each rule. A lighter color in the heatmap plotted in Figure 4c means higher compliance, while a darker color means less compliance. “Hydroxanthone”, “*O*-heterocyclic”, “simple”, and “prenylated” xanthones meet most of the criteria defined by classical rules, except for the Gleeson rules. On the contrary, “complex” xanthones violate at least one criterion in all the considered rules (Figure 4c). Among the classical rules, the Gleeson rules [13] were the best to discriminate the different types of xanthones. “Simple” and “hydroxanthones” obey most of the criteria, “prenylated” obey just some, and “complex” xanthones do not obey the generality of Gleeson’s proposed criteria.

Classical rules have many exceptions, and there are many examples, namely among NP or NP-inspired, of successful drugs that violate them [26]. Quantitative estimate of drug-likeness (QED) is an alternative way for assessing drug-likeness. QED index is generated considering eight properties, namely MW, log P, TPSA, RB, number of hydrogen donors and acceptors, number of aromatic rings, and number of alerts for undesirable substructures [14]. Compared with classical drug-likeness rules, the QED method is more flexible because it does not use cutoffs but a continuous score index of drug-likeness. When all properties are unfavorable, the QED index is 0, and when all properties are favorable, the score is 1 [14]. The obtained QED indexes for marine xanthones clearly differentiate the distinct types (Table S2, Figure 4c,d), enabling sorting the marine xanthones in the following ascending order of drug-likeness: “complex”, “prenylated”, “simple”, “*O*-heterocyclic”, and “hydroxanthones”. The low drug-likeness of “complex” marine xanthones is related to their high MW (Figure 3a) and low solubility (Figure 3f), while the drug-likeness of “hydroxanthones” is ascribed to their low lipophilicity (Figure 3d) and good water solubility (Figure 3f).

**Figure 5:**
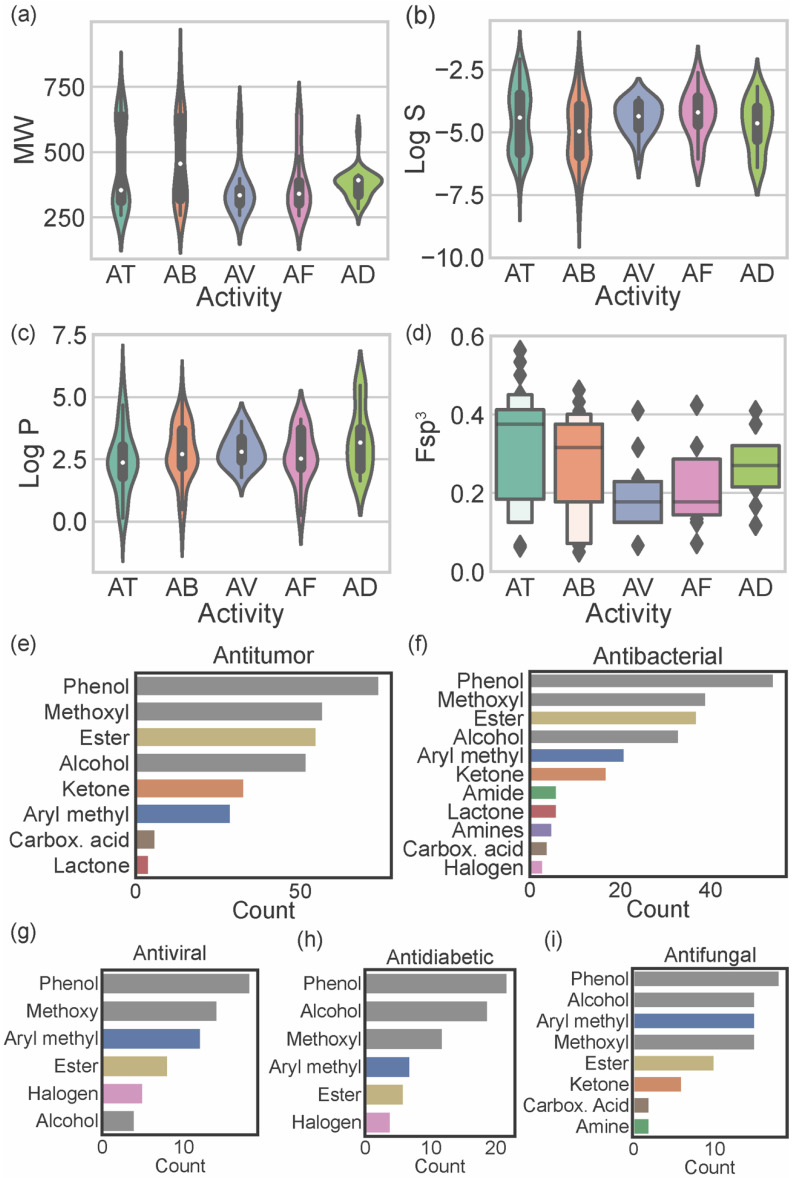
Distribution of MW (**a**); log S (**b**); log P (**c**); and fraction of sp^3^ carbons (**d**) accordingly to the biological activity reported for marine xanthones: antitumor (AT), antibacterial (AB), antiviral (AV), antifungal (AF), and antidiabetic (AD). Analysis of the functional group frequently found on antitumor (**e**); antibacterial (**f**); antiviral (**g**); antifungal (**h**), and antidiabetic (**i**) marine xanthones.

Considering the reported biological activities of marine xanthones, the relationship between these and the molecular descriptors was established. Figure 5a–d displays the relationship of the most relevant molecular descriptors (MW, log S, log P, and Fsp^3^) with the most representative biological activities (antitumor, antibacterial, antifungal, antiviral, and antidiabetic). As the number of xanthones reported for each biological activity is different, the results should only be compared when the number of reported molecules is similar (antitumor vs. antibacterial and antifungal vs. antiviral vs. antidiabetic).

Antitumor marine xanthones have a bimodal distribution of MW with 2 subsets of different sized compounds (one mode of 314.3 g mol^−1^ and another mode of 636.6 g mol^−1^) (Figure 5a). The presence of a bimodal distribution is also observed for the solubility of antitumor marine xanthones (modes of −3.45 and −5.66) (Figure 5b), which is not surprising considering that log S and MW are strictly correlated (Figure 3k). However, log P have a unimodal distribution with a mode of 2.33 (Figure 5c). Similarly, antibacterial marine xanthones also showed a bimodal distribution of MW (modes of 336.3 and 628.6 314.3 g mol^−1^) and log S values (modes of −4.23 and −5.84) (Figure 5a,b) and a unimodal distribution of log P values (mode of 2.37) (Figure 5c). The features of the large-sized subset of antitumor/antibacterial marine xanthone, i.e., “obese” molecules that apparently violate drug-likeness but with a suitable log P value, is a trait of NPs molecules. Despite being often cited as exceptions to classical drug-likeness rules, NP molecules largely comply in terms of log P [27]. This is attributed to the way in which natural evolution took place, producing bioactive compounds that retain low hydrophobicity, even for molecules with high MW [27]. The major difference between the physicochemical properties of antitumor and antibacterial xanthones was in terms of carbon saturation. Antibacterial marine xanthones have lower and more dispersed Fsp^3^ values than antitumor xanthones (median value of 0.38 and 0.32 for antibacterial and antitumor, respectively), raising the hypothesis that more rigidity might be an important aspect for the antibacterial activity (Figure 5d).

Antiviral, antifungal, and antidiabetic marine xanthones presented a unimodal distribution of MW values representing only one set of similar-sized compounds (mode values ranged from 334.7 to 394.5 g mol^−1^) (Figure 5a). Antidiabetic xanthones have a narrower probability distribution of the MW values (Figure 5a), and antiviral xanthones have a narrower dispersion of log S probability distribution (Figure 5b) and of log P probability distribution (Figure 5c). Antiviral and antifungal marine xanthones tend to be quite rigid molecules as they show the lowest Fsp^3^ values (median values of 0.18, Figure 5d). Antiviral, antifungal, and antidiabetic marine fulfill the limited preconized by the drug-likeness filters independently of the descriptor.

The most representative heteroatom in marine xanthones is the oxygen atom, distributed by phenols (present in 96.5% of the reported marine xanthones), methoxy groups (67.8%), alcohols (63.7%), esters (58.5%), ketones (26.9%), and carboxylic acids (7.0%) (Table S3). Other heteroatoms, different from oxygen, present in marine xanthones are halogens (9.4%), mainly the chloride atom, followed by the nitrogen atom, distributed by amines (10 marine xanthones) and amides (8 marine xanthones). The distribution of chemical functional groups by biological activities of marine xanthones was analyzed (Figure 5e–i). The most prevalent functional groups in antitumor, antibacterial, and antiviral xanthones are phenolic and methoxy groups (grey bars on Figure 5e–g). The most prevalent functional groups in antidiabetic and antifungal are phenolic and alcohol groups (grey bars on Figure 5h,i). Esters are very common in xanthones independent of biological activity. Ketones and aryl methyl groups are common in antitumor, antibacterial, and antifungal marine xanthones (Figure 5e,f,i). Halogens are present in some antiviral and antidiabetic xanthones. Nitrogen-containing groups, like amide and amines, are present in antibacterial and antifungal xanthones. Amine groups, which are protonated at physiological pH, could be important for the anti-infective activity of the marine xanthones bearing this group.

## 4. Biological Activities of Marine Xanthones

**Figure 6:**
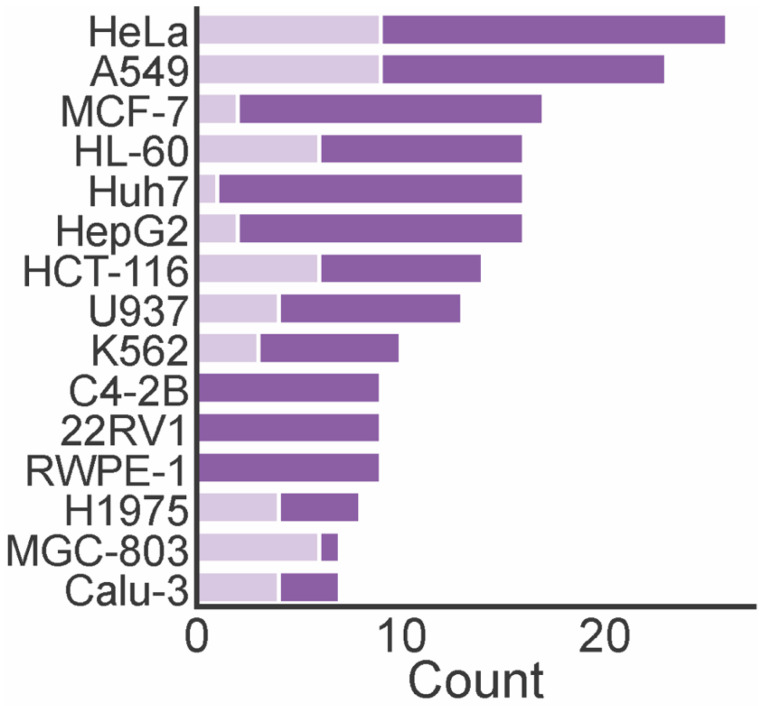
The number of antitumor marine xanthone and the most frequently assayed tumor cell lines. Dark purple bar represents the total count of the assayed xanthones. Light purple bar represents the count of xanthones with IC_50_ lower than 10 µM.

A total of 74 marine xanthones described in literature were evaluated for antitumor activity, measuring their growth inhibitory activity in different tumor cell lines (Table 1). The cervical carcinoma cell line (HeLA), human lung carcinoma (A549), human breast adenocarcinoma cell line (MCF-7), and human leukemia cell line (HL-60) were the most used tumor cell lines in the biological assays (Figure 6). The number of xanthones with a half maximum inhibitory concentration (IC_50_) lower than 10 µM varied depending on the tested cell lines. For instance, the number of xanthones screened against HL-60, MCF-7, and Huh7 cells was almost the same, but the number of most potent xanthones was higher identified against HL-60 cells. None of the marine xanthones assayed against C4-2B, 22RV1, and RWPE-1 presented an IC_50_ lower than 10 µM, while for MGC-803, the most part presented an IC_50_ lower than 10 µM.

**Figure 7:**
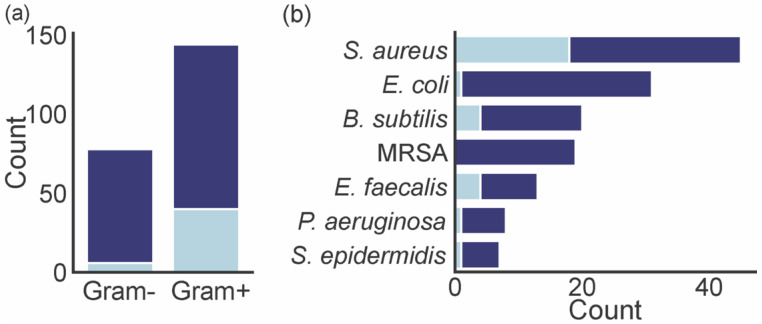
(**a**) The number of antibacterial marine xanthones evaluated against Gram-positive and Gram-negative bacteria. Dark blue bar represents the total count of the assayed xanthones. Light blue bar represents the count of xanthones with MIC lower than 4 µg mL^−1^. (**b**) The bacteria that were assayed for antibacterial activity. Dark blue bar represents the total count of the assayed xanthones. Light blue bar represents the count of xanthones with MIC lower than 4 µg mL^−1^.

A total of 54 marine xanthones described in literature were evaluated for antibacterial activity, measuring the growth inhibitory activity of different bacteria (Table 2). Gram-positive bacteria were more exploited (144 assessments) than Gram-negative (78 assessments) (Figure 7a). The number of xanthones with a minimum inhibitory concentration (MIC) lower than 4 µg mL^−1^ varied depending on the tested bacteria. Marine xanthones revealed a great selectivity for growth inhibition of Gram-positive bacteria as the percentage of marine xanthones with MIC lower than 4 is significantly higher in these types of bacteria (28% Gram-positive vs. 8% Gram-negative). This could be ascribed to a target potentially involving the peptidoglycan layer that is present in Gram-positive bacteria and absent in Gram-negative bacteria. The majority of marine xanthones were evaluated against *S. aureus* and *E. coli* (Figure 7b). Marine xanthones were particularly active against *S. aureus*, *B. subtilis*, *E. faecalis*, which are all Gram-positive bacteria.

A total of 21 marine xanthones were evaluated for the antifungal activity against 17 different fungi, measuring the growth inhibitory activity of different fungi (Table 3). Among the evaluated fungi, *Fusarium* (12 xanthones), *Colletotrichum* (8 xanthones), *Candida* (5 xanthones), and *Microbotryum* (4 xanthones) were the most frequent genus.

A total of 18 marine xanthones were evaluated against different viral targets of H1N1 (10 xanthones), HSV-2 (10 xanthones), HSV-1 (7 xanthones), HIV-1 (3 xanthones), EV71 (2 xanthones), H3N2 (2 xanthones), TMV (1 xanthone) (Table 4).

A total of 19 marine xanthones were evaluated for antidiabetic activity using two different approaches: the assessment of α-glucosidase or protein tyrosine phosphatases inhibition activity and the assessment of the induction of the pancreatic β-cells proliferation in a zeafish model (Table 5).

A total of 9 marine xanthones were evaluated for anti-oxidant activity through the DPPH assay and ABTS or trolox equivalent antioxidant capacity (TEAC) assay (Table 6).

A total of 8 marine xanthones were evaluated for anti-inflammatory activity by measuring the inhibitory activity against cyclooxygenase (COX), by measuring the inhibition of inflammatory response induced by nitric oxide (NO) and NF-κΒ (factor nuclear kappa B), and by measuring the decrease in IL-6 cytokine production on LPS-stimulated macrophages (Table 7).

The remaining biological activities were classified as miscellaneous (Table 8). Seven marine xanthones were evaluated for their immunosuppressive activity through the assessment of the inhibition of proliferation of mouse splenic lymphocytes stimulated with Con-A and LPS. One xanthone was evaluated for its anti-Alzheimer activity through the assessment of acetylcholinesterase inhibition. Three marine xanthones were evaluated for their antiprotozoal activity against *Trypanosoma brucei*, *Trypanosoma cruzi*, *Leshnmania donovani,* and *Plamodium falciparum*. Nine marine xanthones were evaluated for their aquatic pathogens biocide activity against *Vibrio* sp.

## 5. Conclusions

As far as we know, 169 bioactive marine xanthone derivatives were reported in the literature up to 2021. They were isolated from microorganisms, mainly from *Aspergillus* sp., which normally live in an endophytic relationship with microorganisms (e.g., algae, sponge, mangrove, among others).

The chemical space occupied by bioactive marine xanthones was described through molecular descriptors. For each structural category, the distribution of the MW, Fsp3, number of RB, Log P, TPSA, and Log S values were described and analyzed. The descriptors were framed accordingly to the NP and drug-likeness concepts. Among the different structural categories of xanthones, “hydroxanthones” and “*O*-heterocyclic” xanthones are those that better resemble NPs and the ones that better fulfill the drug-likeness criteria. Therefore, hydroxanthones” and “*O*-heterocyclic” xanthones represent the most promising starting point for a hit-to-lead expansion.

In terms of biological activities, a total of 13 different activities were reported for marine xanthones. The antitumor and antibacterial activities were the most predominant. Potent antitumor marine xanthones (IC_50_ < 10 µM) were identified mostly against HeLa (cervical carcinoma), A549 (non-small cell lung carcinoma), HL-60 (acute myeloid leukemia), HCT-116 (colon carcinoma), and MCG-803 (Gastric mucinous adenocarcinoma) cells lines. The most potent antibacterial marine xanthones (MIC < 4 µg mL^−1^) were identified predominantly against Gram-positive bacteria, namely against *S. aureus*, *B. subtilis*, and *E. faecalis*.

Xanthones isolated from marine and terrestrial organisms share a similar biosynthetic pathway. However, marine-derived xanthones have not been as exploited as terrestrial-derived xanthones in traditional drug discovery campaigns. The numerous and relevant bioactive xanthones isolated from the marine environment could inspire the development of new drugs. The data concerning this review allow us to go deeper in understanding molecular properties, at different levels, of such an important family of marine NP.

## Figures and Tables

**Table 1:** Antitumor marine xanthones.

Name	Activity	Source	Ref.
Engyodontiumone B (**1**)	U937 (IC_50_ = 55.5 µM); Hela (IC_50_ = 96.1 µM); MCF-7 (IC_50_ = 172.3 µM); HepG2 (IC_50_ = 73.8 µM); Huh7 (IC_50_ ≥ 300 µM)	*Engyodontium album* (DFFSCS02) isolated from sediment collected in the South China Sea	[28]
Sydowinin A (**2**)	U937 (IC_50_ = 75.6 µM); Hela (IC_50_ ≥ 300 µM); MCF-7 (IC_50_ ≥ 300 µM); HepG2 (IC_50_ ≥ 300 µM); Huh7 (IC_50_ ≥ 300 µM)		
Sydowinin B (**3**)	U937 (IC_50_ = 127.0 µM); Hela (IC_50_ ≥ 300 µM); MCF-7 (IC_50_ ≥ 300 µM); HepG2 (IC_50_ ≥ 300 µM); Huh7 (IC_50_ ≥ 300 µM)		
2,6-Dihydroxy-3-methyl-9-oxoxanthene-8-carboxylic acid methyl ester (**4**)	HEp-2 (IC_50_ = 8 µg mL^−1^); HepG2 (IC_50_ = 9 µg mL^−1^)	Endophytic fungus (SK7RN3G1) isolated from mangrove collected in the South China Sea	[29]
Monodictyxanthone (**5**)	Hepa-1c1c7(Cyp1A inhibition (IC_50_ = 34.8 ± 7.4 µM); NAD(P)H:quinone reductase induction (CD ≥ 50 (1.4) µM, IC_50_ ≥ 50 µM)	*Monodictys putredinis* isolated from the inner tissue of a green alga collected at Tenerife	[30]
8-Hydroxy-6-methylxanthone-1-carboxylic acid (**6**)	% Inhibitions on the cell proliferation at 10 μM: 22RV1 (71.3 ± 1.2%); C4-2B (60.7 ± 5.1%); RWPE-1 (19.7 ± 4.9%)	*Cladosporium halotolerans* (GXIMD 02502) isolated from a coral collected in Beibu Gulf	[31]
Methyl 8-hydroxy-6-methyl-9-oxo-9H-xanthene-1- carboxylate (**7**)	% Inhibitions on the cell proliferation at 10 μM: 22RV1 (55.8 ± 3.0%); C4-2B (8.1 ± 20.6%); RWPE-1 (5.3 ± 3.1%)		
Methyl 8-hydroxy-6-(hydroxymethyl)-9-oxo-9H-xanthene-1-carboxylate (**8**)	% Inhibitions on the cell proliferation at 10 μM: 22RV1 (68.1 ± 1.9%); C4-2B (20.2 ± 0.1%); RWPE-1 (19.0 ± 8.5%)		
Vertixanthone (**9**)	% Inhibitions on the cell proliferation at 10 μM: 22RV1 (27.1 ± 6.9%); C4-2B (−0.1 ± 4.6%); RWPE-1 (25.0 ± 7.9%)		
8-(Methoxycarbonyl)-1-hydroxy-9-oxo-9H-xanthene-3-carboxylic acid (**10**)	% Inhibitions on the cell proliferation at 10 μM: 22RV1 (63.9 ± 2.2%); C4-2B (12.2 ± 5.2%); RWPE-1 (27.0 ± 5.1%)		
3,8-Dihydroxy-6-methyl-9-oxo-9H-xanthene-1-carboxylate (**11**)	% Inhibitions on the cell proliferation at 10 μM: 22RV1 (82.1 ± 0.9%); C4-2B (77.7 ± 0.5%); RWPE-1 (11.5 ± 1.5%)		
Conioxanthone A (**12**)	% Inhibitions on the cell proliferation at 10 μM: 22RV1 (36.8 ± 13.3%); C4-2B (3.3 ± 11.3%); RWPE-1 (20.3 ± 9.0%)		
Questin (**13**)	A549 (IC_50_ = 40.0 ± 0.3 µM); HepG2 (IC_50_ = 42.2 ± 0.5 µM); HeLa (IC_50_ = 36.2 ± 0.9 µM)	*Aspergillus sydowii* (C1-S01-A7) collected in the West Pacific Ocean	[32]
Penixanacid A (**14**)	HeLa (IC_50_ = 10.0 µM); BEL-7402 (IC_50_ = 30.6 µM); HEK-293 (IC_50_ = 28.5 µM); HCT-116 (IC_50_ = 19.0 µM); A-549 (IC_50_ = 16.9 µM)	*Penicillium chrysogenum* (HND11-24) isolated from a mangrove	[33]
Norlichexanthone (**17**)	K562 (IC_50_ = 74.6 μM); A549 (IC_50_ = 64.6 μM); Huh-7 (IC_50_ >30 μM); H1975 (IC_50_ = 79.1 μM); MCF-7 (IC_50_ = 56.7 μM); U937 (IC_50_ > 30 μM); BGC823 (IC_50_ = 697.6 μM); HL-60 (IC_50_ > 30 μM); MOLT-4 (IC_50_ = 135.4 μM); Hela (IC_50_ = 7.2 μM)	*Stachybotry* sp. (ZSDS1F1-2) isolated from a sponge collected at Xisha Island	[34]
Yicathin C (**18**)	A549 (IC_50_ = 37.7 ± 0.3 µM)	*Aspergillus sydowii* (C1-S01-A7) collected in the West Pacific Ocean	[32]
	A375-C5 (IC_50_ = 48.70 ± 4.24 µM); MCF-7 (IC_50_ = 98.93 ± 9.83 µM); NCI-H460 (IC_50_ = 79.83 ± 18.45 µM)	*Aspergillus wentii* isolated from *Gymnogongrus flabelliformis* collected at Pingtan Island	[4]
Yicathin B (**19**)	A375-C5 (IC_50_ = 47.70 ± 2.62 µM); MCF-7 (IC_50_ = 73.92 ± 2.28 µM); NCI-H460 (IC_50_ = 86.21 ± 2.30 µM)		
2-Hydroxy-6-formyl-vertixanthone (**23**)	HepG2 (IC_50_ = 32.7 ± 0.9 µM)	*Aspergillus sydowii* (C1-S01-A7) collected in the West Pacific Ocean	[32]
12-*O*-Acetyl-sydowinin A (**24**)	A549 (IC_50_ = 25.2 ± 0.9 µM); HepG2 (IC_50_ = 42.3 ± 0.6 µM); HeLa (IC_50_ = 33.6 ± 0.7 µM)		
Emodin (**25**)	HeLa (IC_50_ = 27.1 ± 0.8 µM)		
Engyodontiumone H (**50**)	U937 (IC_50_ = 4.9 µM); Hela (IC_50_ = 24.8 µM); MCF-7 (IC_50_ = 38.5 µM); HepG2 (IC_50_ = 60.5 µM); Huh7 (IC_50_ = 53.3 µM)	*Engyodontium album* (DFFSCS021) from isolated sediment collected in the South China Sea	[28]
Engyodontiumone C (**51**)	U937 (IC_50_ = 218.4 µM); Hela (IC_50_ ≥ 300 µM); MCF-7 (IC_50_ ≥ 300 µM); HepG2 (IC_50_ ≥ 300 µM); Huh7 (IC_50_ ≥ 300 µM)		
Engyodontiumone D (**52**)	U937 (IC_50_ = 208.6 µM); Hela (IC_50_ ≥ 300 µM); MCF-7 (IC_50_ ≥ 300 µM); HepG2 (IC_50_ ≥ 300 µM); Huh7 (IC_50_ ≥ 300 µM)		
Engyodontiumone E (**53**)	U937 (IC_50_ = 15.9 µM); Hela (IC_50_ = 205.9 µM); MCF-7 (IC_50_ ≥ 300 µM); HepG2 (IC_50_ ≥ 300 µM); Huh7 (IC_50_ ≥ 300 µM)		
Engyodontiumone F (**54**)	U937 (IC_50_ = 192.7 µM); Hela (IC_50_ ≥ 300 µM); MCF-7 (IC_50_ ≥ 300 µM); HepG2 (IC_50_ ≥ 300 µM); Huh7 (IC_50_ ≥ 300 µM)		
Engyodontiumone G (**55**)	U937 (IC_50_ = 287.2 µM); Hela (IC_50_ ≥ 300 µM); MCF-7 (IC_50_ ≥ 300 µM); HepG2 (IC_50_ ≥ 300 µM); Huh7 (IC_50_ ≥ 300 µM)		
Globosuxanthone A (**56**)	HCT-15 (IC_50_ = 10.7 µM); T-cell leukemia Jurkat cells (IC_50_ = 2.3 µM)	*Beauveria bassiana* (TPU942) isolated from a piece of an unidentified sponge collected at Iriomote Island	[35]
Monodictysin A (**57**)	Hepa-1c1c7(Cyp1A inhibition IC_50_ ≥ 50 µM); NAD(P)H:quinone reductase induction (CD = 191.1 µM, IC_50_ ≥ 400 µM)	*Monodictys putredinis* isolated from the inner tissue of a green alga collected at Tenerife	[30]
Monodictysin B (**58**)	Hepa-1c1c7(Cyp1A inhibition IC_50_ = 23.3 ± 3.9 µM); NAD(P)H:quinone reductase induction (CD = 12.0 ± 4.8 µM, IC_50_ ≥ 50 µM)		
Monodictysin C (**59**)	Hepa-1c1c7(Cyp1A inhibition IC_50_ = 3.0 ± 0.7 µM); NAD(P)H:quinone reductase induction (CD = 12.8 ± 2.6 µM, IC_50_ ≥ 50 µM)		
α-Diversonolic ester (**60**)	% Inhibitions on the cell proliferation at 10 μM: 22RV1 (28.8 ± 10.3%); C4-2B (12.9 ± 12.6%); RWPE-1 (24.3 ± 3.3%)	*Cladosporium halotolerans* (GXIMD 02502) isolated from a coral collected in Beibu Gulf	[31]
β-Diversonolic ester (**61**)	% Inhibitions on the cell proliferation at 10 μM: 22RV1 (40.2 ± 1.5%); C4-2B (2.8 ± 2.2%); RWPE-1 (10.3 ± 3.8%)		
Penixanthone A (**62**)	Weak cytotoxicity against H1975, MCF-7, K562, HL7702 at concentration of 30 μM.	*Penicillium* sp. (SYFz-1) isolated from a mangrove sample	[36]
AGI-B4 (**64**)	K562 (IC_50_ = 6.97 µM); MCF-7 (IC_50_ = 11.7 µM); HeLa (IC_50_ = 1.39 µM); DU145 (IC_50_ = 2.69 µM); U937 (IC_50_ = 0.463 µM); H1975 (IC_50_ = 8.53 µM); SGC-7901 (IC_50_ = 9.43 µM); A549 (IC_50_ = 7.01 µM); MOLT-4 (IC_50_ = 5.26 µM); HL-60 (IC_50_ = 6.20 µM)	*Aspergillus* sp. (SCSIO Ind09F01)	[37]
	U937 (IC_50_ = 8.8 µM); Hela (IC_50_ = 60.0 µM); MCF-7 (IC_50_ = 102.2 µM); HepG2 (IC_50_ = 52.7 µM); Huh7 (IC_50_ = 133.3 µM)	*Engyodontium album* (DFFSCS021) isolated from sediment collected in the South China Sea	[28]
	L5178Y (IC_50_ = 1.5 µM)	*Scopulariopsis* sp. isolated from solid rice cultures obtained from the Red Sea hard coral *Stylophora* sp.	[38]
Versicone G (**67**)	NB_4_ (IC_50_ = 15.6 µM); HL-60 (IC_50_ = 21.7 µM); HeLa (IC_50_ = 16.9 µM)	*Aspergillus versicolor* (HDN11-84) isolated from mangrove	[39]
Paeciloxanthone (**68**)	HepG2 (IC_50_ = 1.08 µg mL^−1^)	*Paecilomyces* sp. isolated from mangrove collected in the Taiwan Strait	[40]
Chaetoxanthone A (**78**)	L6-cells (IC_50_ = 59.1 μg/mL)	*Chaetomium* sp. isolated from the Greek alga collected at Santorini Island.	[41]
Chaetoxanthone B (**79**)	L6-cells (IC_50_ > 90 μg/mL)		
Chaetoxanthone C (**80**)	L6-cells (IC_50_ = 46.7 μg/mL)		
Sterigmatocystin (**81**)	Bel-7402 (IC_50_ = 96.53 µg mL^−1^); NCIH-460 (IC_50_ = 72.52 µg mL^−1^)	Fungal strain (isolate 1850) isolated from a leaf of *Kandelia candel* collected in Hong Kong	[42]
	A-549 (IC_50_ = 1.86 µg mL^−1^); SK-OV-3 (IC_50_ = 2.53 µg mL^−1^); SK-MEL-2 (IC_50_ = 1.22 µg mL^−1^); XF- 498 (IC_50_ = 2.75 µg mL^−1^); HCT-15 (IC_50_ = 4.61 µg mL^−1^)	*Aspergillus versicolor* isolated from *Petrosia* sp.	[43]
	A-549 (IC_50_ = 11.25 μg mL^−1^); SK-OV-3 (IC_50_ = 17.36 μg mL^−1^); SK-MEL-2 (IC_50_ = 14.33 μg mL^−1^); XF-498 (IC_50_ = 15.12 μg mL^−1^); HCT-15 (IC_50_ ≥ 30 μg mL^−1^)	*Aspergillus versicolor*	[44]
Aspergixanthone A (**82**)	A-549 (IC_50_ = 1.8 µM)	*Aspergillus* sp. (ZA-01) isolated from sediment collected in the Bohai Sea	[45]
Aspergixanthone C (**83**)	MDA-MB-231 (IC_50_ = 3.3 µM); MCF-7 (IC_50_ = 2.8 µM); MGC-803 (IC_50_ = 3.6 µM); HeLa (IC_50_ = 2.9 µM); A-549 (IC_50_ = 3.2 µM)		
Aspergixanthone F (**84**)	MDA-MB-231 (IC_50_ = 9.8 µM); MCF-7 (IC_50_ = 2.7 µM); MGC-803 (IC_50_ = 3.6 µM); HeLa (IC_50_ = 1.7 µM); A-549 (IC_50_ = 1.1 µM)		
5-Methoxysterigmatocystin (**85**)	A-549 (IC_50_ = 3.86 µM); HL-60 (IC_50_ = 5.32 µM)	*Aspergillus versicolor*	[46]
Epiremisporine B (**121**)	K562 (IC_50_ = 16.6 µM); MCF-7(IC_50_ = 16.3 µM); SGC7901 (IC_50_ = 15.8 µM)	*Penicillium* sp. (SCSIO Ind16F01) isolated from a deep-sea sediment collected in the Indian Ocean	[47]
Dicerandrol C (**122**)	MDA-MB-435 (IC_50_ = 44.10 ± 2.45 µM); HCT-116 (IC_50_ = 42.63 ± 2.90 µM); Calu-3 (IC_50_ = 36.52 ± 3.32 µM); Huh7 (IC_50_ ≥ 50 µM); MCF-10A (IC_50_ = 33.05 ± 2.74 µM)	*Phomopsis* sp. (HNY29-2B) isolated from *Acanthus ilicifolius* collected in the South China Sea	[48]
Secalonic acid D (**123**)	PANC-1 Glucose (−) (IC_50_ = 0.6 µM); PANC-1 Glucose (+) (IC_50_ ≥ 1000 µM)	*Penicilliumoxalicum* (16A08-1-1) isolated from a sponge collected at Pramuka Island	[49]
	U87 MG (IC_50_ = 5.64 µM); NCI-H1650 (IC_50_ = 4.93 µM); HT29 (IC_50_ = 1.46 µM)A498 (IC_50_ = 8.88 µM); HL-60 (IC_50_ = 0.41 µM)	*Penicillium chrysogenum* (HLS111) isolated from a sponge	[50]
	SK- HEP (IC_50_ = 1.504 µM); Hela (IC_50_ = 1.322 µM); A549 (IC_50_ = 1.625 µM); SK-MES-1 (IC_50_ = 1.314 µM); SPC-A1 (IC_50_ = 1.679 µM); 95D (IC_50_ = 1.003 µM); Jeko-1 (IC_50_ = 0.915 µM); Raji (IC_50_ = 0.955 µM); U937 (IC_50_ = 1.119 µM); A375 (IC_50_ = 1.598 µM); HFF (IC_50_ = 24.1 µM); H22 (IC_50_ = 1.007µM)	*Penicilliumoxalicum* isolated from sediments collected on the southeast coastal region of China	[51]
Versixanthone G (**124**)	HL-60 (IC_50_ = 13.4 µM); K562 (IC_50_ = 20.9 µM); A549 (IC_50_ = 17.8 µM); H1975 (IC_50_ = 9.8 µM); MGC803 (IC_50_ = 4.6 µM); HEK293 (IC_50_ ≥ 50 µM); HO-8910 (IC_50_ = 9.6 µM); HCT-116 (IC_50_ = 16.2 µM)	*Aspergillus versicolor* isolated from mangrove	[52]
Versixanthone H (**125**)	HL-60 (IC_50_ = 6.9 µM); K562 (IC_50_ = 22.1 µM); A549 (IC_50_ = 19.2 µM); H1975 (IC_50_ = 5.3 µM); MGC803 (IC_50_ = 6.2 µM); HEK293 (IC_50_ ≥ 50 µM); HO-8910 (IC_50_ = 6.9 µM); HCT-116 (IC_50_ = 15.2 µM)		
Versixanthone I (**126**)	HL-60 (IC_50_ = 27.8 µM); K562 (IC_50_ ≥ 50.0 µM); A549 (IC_50_ ≥ 50.0 µM); H1975 (IC_50_ ≥ 50.0 µM); HEK293 (IC_50_ ≥ 50 µM); HO-8910 (IC_50_ ≥ 50.0 µM); HCT-116 (IC_50_ ≥ 50.0 µM)		
Versixanthone J (**127**)	HL-60 (IC_50_ = 47.3 µM); K562 (IC_50_ ≥ 50.0 µM); A549 (IC_50_ ≥ 50.0 µM); H1975 (IC_50_ ≥ 50.0 µM); HEK293 (IC_50_ ≥ 50 µM); HO-8910 (IC_50_ ≥ 50.0 µM); HCT-116 (IC_50_ ≥ 50.0 µM)		
Versixanthone K (**128**)	HL-60 (IC_50_ = 49.5 µM); K562 (IC_50_ ≥ 50.0 µM); A549 (IC_50_ ≥ 50.0µM); H1975 (IC_50_ = 49.5 µM); MGC803 (IC_50_ ≥ 50.0µM); HEK293 (IC_50_ ≥ 50 µM); HO-8910 (IC_50_ ≥ 50.0 µM); HCT-116 (IC_50_ ≥ 50.0 µM)		
Versixanthone L (**129**)	HL-60 (IC_50_ = 0.5 µM); K562 (IC_50_ = 1.1 µM); A549 (IC_50_ = 1.6 µM); MGC803 (IC_50_ = 1.1 µM); HO-8910 (IC_50_ = 1.5 µM); HCT-116 (IC_50_ = 1.2 µM)		
Versixanthone M (**130**)	HL-60 (IC_50_ = 0.9 µM); K562 (IC_50_ = 0.4 µM); A549 (IC_50_ = 11.7 µM); H1975 (IC_50_ = 3.5 µM); MGC803 (IC_50_ = 0.9 µM); HO-8910 (IC_50_ = 1.4 µM); line HCT-116 (IC_50_ = 0.5 µM)		
Citreamicin ε A (**131**)	HeLa (IC_50_ = 0.032 ± 0.0062 µM); HepG2 (IC_50_ = 0.079 ± 0.031 µM)	*Streptomyces caelestis* collected on the coastal water of the Red Sea	[53]
Citreamicin ε B (**132**)	HeLa (IC_50_ = 0.031 ± 0.0081 µM); HepG2 (IC_50_ = 0.10 ± 0.0053 µM)		
Acredinone C (**133**)	Inhibited the RANKL- induced formation of TRAP^+^-MNCs in a dose-dependent manner without any cytotoxicity up to 10 μM	*Acremonium* sp. isolated from the inner tissue of *Suberites japonicas*	[54]
Phomolactonexanthone A (**134**)	Calu-3 (IC_50_ = 43.45 ± 2.51 µM)	*Phomopsis* sp. (HNY29-2B) isolated from *Acanthus ilicifolius* collected in the South China Sea	[48]
Deacetylphomoxanthone C (**135**)	HCT-116 (IC_50_ = 44.06 ± 3.29 µM); Calu-3 (IC_50_ = 43.35 ± 2.09µM)		
Dicerandrol A (**136**)	MDA-MB-435 (IC_50_ = 3.03 ± 0.12 µM); HCT-116 (IC_50_ = 2.64 ± 0.03 µM); Calu-3 (IC_50_ = 1.76 ± 0.02 µM); Huh7 (IC_50_ = 4.19 ± 0.08 µM); MCF-10A (IC_50_ = 28.32 ± 3.57µM)		
Dicerandrol B (**137**)	MDA-MB-435 (IC_50_ = 8.65 ± 0.66 µM); HCT-116 (IC_50_ = 3.94 ± 0.39µM); Calu-3 (IC_50_ = 4.10 ± 0.08 µM); Huh7 (IC_50_ = 30.37 ± 1.10 µM); MCF-10A (IC_50_ = 8.14 ± 1.27 µM)		
Deacetylphomoxanthone B (**138**)	MDA-MB-435 (IC_50_ = 14.40 ± 1.18 µM); HCT-116 (IC_50_ = 7.12 ± 0.70 µM); Calu-3 (IC_50_ = 4.14 ± 0.02 µM); Huh7 (IC_50_ = 29.20 ± 1.19 µM)		
Penexanthone A (**139**)	MDA-MB-435 (IC_50_ = 7.90 ± 0.58µM); HCT-116 (IC_50_ = 6.92 ± 0.38 µM); Calu-3 (IC_50_ = 6.44 ± 0.86 µM); Huh7 (IC_50_ = 42.82 ± 3.58 µM); MCF-10A (IC_50_ = 16.13 ± 1.57 µM)		
4,4′-bond Secalonic acid D (**140**)	SK- HEP (IC_50_ = 1.342 µM); Hela (IC_50_ = 0.827 µM); A549 (IC_50_ = 1.353 µM); SK-MES-1 (IC_50_ = 0.640 µM); SPC-A1 (IC_50_ = 1.205 µM); 95D (IC_50_ = 0.978 µM); Jeko-1 (IC_50_ = 0.705 µM); Raji (IC_50_ = 0.484 µM); U937 (IC_50_ = 0.960 µM); A375 (IC_50_ = 1.085 µM); HFF (IC_50_ = 26.6 µM); H22 (IC_50_ = 1.211 µM)	*Penicilliumoxalicum* isolated from sediments collected on the southeast coastal region of China	[51]
Phomoxanthone A (**141**)	HL-60 (cytotoxic at 0.1 to 0.01 μg mL^−1^)	*Phomopsis longicolla* isolated from *Bostrychia radicans*	[55]
JBIR-97 (**142**/**143**)	HeLa (IC_50_ = 11 µM); ACC-MESO-1 (IC_50_ = 31 µM)	*Tritirachium* sp. (SpB081112MEf2) isolated from *Pseudoceratina purpurea* collected at Ishigaki Island	[56]
JBIR-98 (**142**/**143**)	HeLa (IC_50_ = 17 µM); ACC-MESO-1 (IC_50_ = 63 µM)		
JBIR-99 (**144**)	HeLa (IC_50_ = 17 µM); ACC-MESO-1 (IC_50_ = 59 µM)		
Buanmycin (**156**)	A549 (IC_50_ = 1.7 µM); HCT116 (IC_50_ = 0.9 µM); SNU638 (IC_50_ = 0.8 µM); SK-HEP1 (IC_50_ = 1.9 µM); MDA-MB231 (IC_50_ = 1.2 µM)	*Streptomyces* sp. isolated from a tidal mudflat collected in Buan	[57]
	A549 (IC_50_ = 0.8 µM); HeLa (IC_50_ = 0.9 µM)	*Streptomyces* sp. (HGMA004) isolated from a mudflat collected at Uki	[58]
Chrysoxanthone A (**162**)	U87 MG (IC_50_ = 22.6 µM); NCI-H1650 (IC_50_ = 42.2 µM); HT29 (IC_50_ = 41.8 µM); A498 (IC_50_ = 28.5 µM); HL-60 (IC_50_ = 37.2 µM)	*Penicillium chrysogenum* (HLS111) isolated from a sponge	[50]
Chrysoxanthone B (**163**)	U87 MG (IC_50_ ≥ 50 µM); NCI-H1650 (IC_50_ ≥ 50 µM); HT29 (IC_50_ = 30.8 µM); A498 (IC_50_ ≥ 50 µM); HL-60 (IC_50_ = 16.2 µM)		
Chrysoxanthone C (**164**)	U87 MG (IC_50_ = 47.0 µM); NCI-H1650 (IC_50_ ≥ 50 µM); HT29 (IC_50_ = 43.2 µM); A498 (IC_50_ ≥ 50 µM); HL-60 (IC_50_ = 22.7 µM)		
Ukixanthomycin A (**165**)	A549 (IC_50_ ≥ 200 µM); HeLa (IC_50_ ≥ 200 µM)	*Streptomyces* sp. (HGMA004) isolated from a mudflat collected at Uki	[58]

IC_50_: half maximum inhibitory concentration; CD: concentration required to double quinone reductase specific activity.

**Table 2:** Antibacterial marine xanthone.

Name	Activity	Source	Ref.
Sydowinin B (**3**)	*V. rotiferianus* (MCCC E385) (MIC = 32.6 ± 1.1 µg mL^−1^)	*Aspergillus sydowii* (C1-S01-A7) collected in the West Pacific Ocean	[32]
1,4,7-Trihydroxy-6-methylxanthone (**15**)	*E. coli* (MIC = 32 μg mL^−1^); *P. aeruginosa* (MIC = 32 μg mL^−1^); *S. aureus* (MIC > 64 μg mL^−1^); *V. alginolyticus* (MIC = 32 μg mL^−1^); *V. harveyi* (MIC = 32 μg mL^−1^); *V. parahaemolyticus* (MIC = 32 μg mL^−1^)	*Talaromyces islandicus* (EN-501) isolated from *Laurencia okamurai*	[59]
1,4,5-Trihydroxy-2-methylxanthone (**16**)	*E. coli* (MIC = 4 μg mL^−1^); *P. aeruginosa* (MIC = 4 μg mL^−1^); *S. aureus* (MIC = 8 μg mL^−1^); *V. alginolyticus* (MIC = 4 μg mL^−1^); *V. harveyi* (MIC = 8 μg mL^−1^); *V. parahaemolyticus* (MIC = 4 μg mL^−1^)		
Norlichexanthone (**17**)	*S. aureus* (ATCC 27154) (MIC = 12.5 µg mL^−1^); *E. coli* (ATCC 25922) (MIC > 100 µg mL^−1^); *S. ventriculi* (ATCC 29068) (MIC = 25.0 µg mL^−1^); *P. aeruginosa* (ATCC 25668) (MIC = 25.0 µg mL^−1^)	*Talaromyces* sp. (ZH-154) collected in the South China Sea	[60]
Yicathin C (**18**)	*E. coli* (zone of inhibition 12.0 mm); *S. aureus* (zone of inhibition 7.5 mm)	*Aspergillus wentii* isolated from *Gymnogongrus flabelliformis* collected at Pingtan Island	[61]
Yicathin B (**19**)	*E. coli* (zone of inhibition 9 mm)		
Fischexanthone (**20**)	*E. coli* (MIC > 1265.82 µM); *S. aureus* (MIC > 1265.82 µM)	*Alternaria* sp. (R6) isolated from mangrove collected at Leizhou peninsula	[62]
Methyl (2-chloro-l,6-dihydroxy-3-methylxanthone)-8-carboxylate (**21**)	*S. aureus* (ATCC43300) (MIC = 6.25 μg mL^−1^); *S. aureus* (ATCC29213) (MIC = 6.25 μg mL^−1^); *S. aureus* (ATCC33591) (MIC = 3.13 μg mL^−1^); *S. aureus* (ATCC25923) (MIC = 3.13 μg mL^−1^); *E. faecalis* (ATCC51299) (MIC = >100 μg mL^−1^); *E. faecium* (ATCC35667) (MIC = >100 μg mL^−1^); *V. parahaemolyticus* (ATCC17802) (MIC > 100 μg mL^−1^)	*Aspergillus flavipes* (DL-11) isolated from coastal sediment collected in Dalian	[63]
Methyl (4- chloro-l,6-dihydroxy-3-methylxanthone)-8-carboxylate (**22**)	*S. aureus* (ATCC43300) (MIC = 3.13 μg mL^−1^); *S. aureus* (ATCC29213) (MIC = 3.13 μg mL^−1^); *S. aureus* (ATCC33591) (MIC = 1.56 μg mL^−1^); *S. aureus* (ATCC25923) (MIC = 3.13 μg mL^−1^); *E. faecalis* (ATCC51299) (MIC 25 μg mL^−1^); *E. faecium* (ATCC35667) (MIC 50 μg mL^−1^); *V. parahaemolyticus* (ATCC17802) (MIC > 100 μg mL^−1^)		
2-Hydroxy-6-formyl-vertixanthone (**23**)	MRSA (ATCC 43300) (MIC = 16.3 ± 0.9 µg mL^−1^); MRSA (CGMCC 1.12409) (MIC = 16.1 ± 0.5 µg mL^−1^)	*Aspergillus sydowii* (C1-S01-A7) collected in the West Pacific Ocean	[32]
12-*O*-Acetyl-sydowinin A (**24**)	MRSA (ATCC 43300) (MIC = 32.6 ± 0.8 µg mL^−1^); MRSA (CGMCC 1.12409) (MIC = 31.8 ± 0.8 µg mL^−1^)		
Emodin (**25**)	*V. vulnificus* (MCCC E1758) (MIC = 16.1 ± 0.7 µg mL^−1^); MRSA (ATCC 43300) (MIC = 15.4 ± 0.3 µg mL^−1^); MRSA (CGMCC 1.12409) (MIC = 15.7 ± 0.5 µg mL^−1^)		
Aspergillusone A (**26**)	MRSA (ATCC 43300) (MIC = 32.2 ± 0.3 µg mL^−1^); MRSA (CGMCC 1.12409) (MIC = 32.4 ± 0.1 µg mL^−1^)		
	BCG (*M. bovis Pasteur* 1173P2) (MIC = 20 µg mL^−1^)	*Aspergillus versicolor* (MF160003)	[64]
Chalaniline B (**27**)	Percent (%) growth of treated bacteria: *B. subtilis* (ATCC 49343) (67 ± 17%); *S. aureus* (ATCC 25923) (64 ± 14%); MRSA (ATCC BAA-41) (57 ± 8%); MRSA (ATCC BAA-44) (40 ± 2%)	Endophytic ascomycete with *Chalara* sp. (6661)	[65]
Engyodontiumone H (**50**)	*E. coli* (zone of inhibition 13.8 mm); *B. subtilis* (zone of inhibition 16.5 mm)	*Engyodontium album* (DFFSCS02) isolated from a sediment collected in the South China Sea	[28]
	*E. coli* (MIC = 64 μg mL^−1^); *B. subtilis* (MIC = 32 μg mL^−1^)		
Aspergillusone B (**63**)	*E. coli* (zone of inhibition 11.0 mm); *B. subtilis* (zone of inhibition 14.4 mm)	*Engyodontium album* (DFFSCS02) isolated from a sediment collected in the South China Sea	
	*E. coli* (MIC = 64 μg mL^−1^); *B. subtilis* (MIC = 64 μg mL^−1^)		
AGI-B4 (**64**)	*E. coli* (zone of inhibition 15.8 mm); *B. subtilis* (zone of inhibition 17.5 mm)	*Engyodontium album* (DFFSCS02) isolated from sediment collected in the South China Sea	
	*E. coli* (MIC = 64 μg mL^−1^); *B. subtilis* (MIC = 64 μg mL^−1^)	*Engyodontium album* (DFFSCS02) isolated from sediment collected in the South China Sea	
	*V. vulnificus* (MCCC E1758) (MIC = 32.5 ± 0.4 µg mL^−1^); MRSA (ATCC 43300) (MIC = 32.9 ± 0.3 µg mL^−1^); MRSA (CGMCC 1.12409) (MIC = 16.3 ± 0.5 µg mL^−1^)	Deep sea-derived fungus *Aspergillus sydowii* C1-S01-A7 isolated in the West Pacific Ocean	[32]
Blennolide A (**65**)	*E. coli* (zone of inhibition 7 mm); *B. megaterium* (zone of inhibition 8 mm)	*Blennoria* sp. isolated from *Carpobrotus edulis* collected at Gomera	[66]
Blennolide B (**66**)	*E. coli* (zone of inhibition 8 mm); *B. megaterium* (zone of inhibition 8 mm)		
Paeciloxanthone (**68**)	*E. coli* (zone of inhibition 12 mm)	*Paecilomyces* sp. isolated from a mangrove collected in the Taiwan Strait	[40]
Stergimatocystin (**81**)	*S. aureus* (zone of inhibition 9.0 mm)	*Aspergillus versicolor*	[44]
Hemi-acetal sterigmatocystin (**86**)	*S*. *aureus* (ATCC 6538) (MIC > 100 μg mL^−1^); *B*. *subtilis* (ATCC 6633) (MIC > 100 μg mL^−1^); MRSA (MIC >100 μg mL^−1^); *P. aeruginosa* (ATCC 15692) (MIC > 100 μg mL^−1^)	*Aspergillus versicolor* (MF359) isolated from *Hymeniacidon perleve* collected in the Bohai Sea	[67]
Acyl-hemiacetal sterigmatocystin (**87**)	*S*. *aureus* (ATCC 6538) (MIC > 100 μg mL^−1^); *B*. *subtilis* (ATCC 6633) (MIC > 100 μg mL^−1^); MRSA (MIC > 100 μg/mL); *P. aeruginosa* (ATCC 15692) (MIC > 100 μg mL^−1^)		
5-Methoxydihydrosterigmatocystin (**88**)	*S*. *aureus* (ATCC 6538) (MIC = 12.5 μg mL^−1^); *B*. *subtilis* (ATCC 6633) (MIC = 3.125 μg mL^−1^); MRSA (MIC > 100 μg mL^−1^); *P. aeruginosa* (ATCC 15692) (MIC > 100 μg mL^−1^)		
Emerixanthone E (**89**)	*E*. *coli* (ATCC 29922); *K*. *pneumoniae* (ATCC 13883); *S*. *aureus* (ATCC 29213), *E*. *faecalis* (ATCC 29212); *A*. *baumannii* (ATCC 19606); *A*. *hydrophila* (ATCC 7966): Diameters of the inhibition zones ranged between 9 and 11 mm	*Emericella* sp. collected in the South China Sea	[68]
Emerixanthone A (**90**)	*E. coli* (ATCC 29922); *K. pneumoniae* (ATCC 13883); *S. aureus* (ATCC 29213); *E. faecalis* (ATCC 29212); *A. baumannii* (ATCC 19606); *A. hydrophila* (ATCC 7966): Diameters of inhibition zones were all 4–6 mm	*Emericella* sp. (SCSIO 05240) collected in the South China Sea	[69]
Emerixanthone C (**91**)	*E. coli* (ATCC 29922); *K. pneumoniae* (ATCC 13883); *S. aureus* (ATCC 29213); *E. faecalis* (ATCC 29212); *A. baumannii* (ATCC 19606); *A. hydrophila* (ATCC 7966): Diameters of inhibition zones were all 4–6 mm		
Varixanthone (**92**)	*E. coli* (MIC = 12.5 µg mL^−1^); *Proteus* sp. (MIC = 12.5 µg mL^−1^); *B. subtilis* (MIC = 12.5 µg mL^−1^); *S. aureus* (MIC = 12.5 µg mL^−1^); *E. faecalis* (MIC = 50 µg mL^−1^)	*Emericella variecolor* (M75-2) was isolated from a *Porifera* sp. collected in the Caribbean Sea	[70]
Oxisterigmatocystin C (**93**)	*S. aureus* (ATCC25923) (MIC < 48 μg mL^−1^)	*Aspergillus* sp. (F40) isolated from *Callyspongia* sp.	[71]
Aspergixanthone G (**94**)	*M. luteus* (MIC = 0.78 µg mL^−1^); *B. anthracis* (MIC = 12.5 µg mL^−1^); *S. typhi* (MIC = 6.13 µg mL^−1^); *E. aerogenes* (MIC = 6.13 µg mL^−1^)	*Aspergillus* sp. (ZA-01) isolated from sediment collected in the Bohai Sea	[45]
Aspergixanthone H (**95**)	*M. luteus* (MIC = 6.13 µg mL^−1^); *B. anthracis* (MIC = 12.5 µg mL^−1^); *S. typhi* (MIC = 6.13 µg mL^−1^); *E. aerogenes* (MIC = 6.13 µg mL^−1^)		
Dicerandrol C (**122**)	*S. aureus* (ATCC 6538) (MIC = 1.33 µM); *S. saprophyticus* (ATCC 15305) (MIC = 2.66 µM)	*Phomopsis longicolla* isolated from *Bostrychia radicans* collected in Brazil	[72]
Secalonic acid D (**123**)	*S. aureus* (ATCC 29,213) (IC_50_ = 7.19 μM); *M. tuberculosis* (IC_50_ = 1.26 μM)	*Aspergillus* sp. (SCSIO XWS03F03) isolated from a sponge	[73]
	*B. subtilis* (MIC = 24.4 µg mL^−1^); *E. coli* J(VC1228) (MIC = 24.4 µg mL^−1^); *M. luteus* (UST950701-006) (MIC = 24.4 µg mL^−1^); *P. nigrifaciens* (UST010620-005) (MIC = 97.5 µg mL^−1^)	*Penicillium* sp. (SCSGAF0023) isolated from *Dichotella gemmacea* collected in the South China Sea	[74]
JBIR-97/98 (**145**)	*S. epidermidis* (IC_50_ = 0.20 ± 0.04 μM); MRSA (IC_50_ = 0.19 ± 0.02 μM); *P. acnes* (IC_50_ = 11.0 ± 1.3 μM)	*Engyodontium album* isolated from *Cacospinga scalaris* collected at the Limski Fjord	[75]
Engyodontochone A (**146**)	*S. epidermidis* (IC_50_ = 0.19 ± 0.04 μM); MRSA (IC_50_ = 0.17 ± 0.02 μM); *P. acnes* (IC_50_ = 13.8 ± 1.7 μM)		
JBIR-99 (**147**)	*S. epidermidis* (IC_50_ = 0.21 ± 0.09 μM); MRSA (IC_50_ = 0.25 ± 0.07μM); *P. acnes* (IC_50_ = 14.1 ± 2.7 μM)		
Engyodontochone B (**148**)	*S. epidermidis* (IC_50_ = 0.22 ± 0.03 μM); *MRSA* (IC_50_ = 0.24 ± 0.04 μM); *P. acnes* (IC_50_ = 11.7 ± 2.4 μM)		
Microluside A (**149**)	*E. faecalis* (JH212) (MIC = 10 μM); *S. aureus* (NCTC 8325) (MIC = 13 μM)	*Micrococcus* sp. (EG45) isolated from *Spheciospongia vagabunda* collected in the Red Sea	[76]
Citreamicin θ A (**150**)	*S. haemolyticus* (MIC = 0.5 μg mL^−1^); *S. aureus* (UST950701-005) (MIC = 1.0 μg mL^−1^); *B. subtillis* (769) (MIC = 0.25 μg mL^−1^); *S. aureus* (ATCC43300) (MIC = 0.25 μg mL^−1^)	*Streptomyces caelestis* collected in the Red Sea	[77]
Citreamicin θ B (**151**)	*S. haemolyticus* (UST950701-004) (MIC = 0.5 μg mL^−1^); *S. aureus* (UST950701-005) (MIC = 1.0 μg mL^−1^); *B. subtilis* (769) (MIC = 0.25 μg mL^−1^); *S. aureus* (ATCC43300) (MIC = 0.25 μg mL^−1^)		
Citreaglycon A (**152**)	*S. haemolyticus* (MIC = 8.0 μg mL^−1^); *S. aureus* (UST950701-005) (MIC = 16 μg mL^−1^); *B. subtilis* (769) (MIC = 8.0 μg mL^−1^); *S. aureus* (ATCC43300) (MIC = 8.0 μg mL^−1^)		
Dehydrocitreaglycon A (**153**)	*S. haemolyticus* (UST950701-004) (MIC = 8.0 μg mL^−1^); *S. aureus* (UST950701-005) (MIC = 16 μg mL^−1^); *B. subtilis* (769) (MIC = 8.0 μg mL^−1^)		
Penicillixanthone A (**154**)	*B. subtilis* (MIC = 24.4 µg mL^−1^); *E. coli* (JVC1228) (MIC = 24.4 µg mL^−1^); *M. luteus* (UST950701- 006) (MIC = 24.4 µg mL^−1^); *P. nigrifaciens* (UST010620-005) (MIC = 97.5 µg mL^−1^)	*Penicillium* sp. (SCSGAF0023) isolated from *Dichotella gemmacea* collected in the South China Sea	[74]
IB-00208 (**155**)	*E. coli* (ATCC 10536) (MIC ≥ 150 nM); *K. pneumonie* (ATCC 29665) (MIC ≥ 150 nM); *P. aeruginosa* (ATCC 10145) (MIC ≥ 150 nM); *B. subtilis* (ATCC 6051) (MIC = 1.4 nM); *S. aureus* (ATCC 6538P) (MIC = 1.4 nM); *M. luteus* (ATCC 9341) (MIC = 0.09 nM)	*Actinomadura* sp. collected at the northern coast of Spain	[78]
Buanmycin (**156**)	*S. aureus* (MIC = 10.5 μM, sortase A inhibition IC_50_ = 43.2 μM); *B. subtilis* (MIC = 0.7 μM); *K. rhizophila* (MIC = 10.5 μM); *S. enterica* (MIC = 0.7 μM); *P. hauseri* (MIC = 21.1 μM)	*Streptomyces* sp. isolated from a tidal mudflat collected in Buan	[57]
	*B. cereus* (IC_50_ = 3.0 µM); *E. coli* (IC_50_ = 6.0 µM)	*Streptomyces* sp. (HGMA004) isolated from a mudflat collected at Uki	[58]
Secalonic acid A (**157**)	*S. aureus* (ATCC 27154) (MIC = 12.5 μg mL^−1^); *E. coli* (ATCC 25922) (MIC = 25 μg mL^−1^); *S. ventriculi* (ATCC 29068) (MIC = 12.5 μg mL^−1^); *P. aeruginosa* (ATCC 25668) (MIC = 12.5 μg mL^−1^)	*Talaromyces* sp. (ZH-154) collected in the South China Sea	[60]
Secalonic acid B (**158**)	*B. subtilis* (MIC = 97.5 µg mL^−1^); *E. coli* (JVC1228) (MIC = 97.5 µg mL^−1^); *M. luteus* (UST950701-006) (MIC = 97.5 µg mL^−1^); *P. nigrifaciens* (UST010620-005) (MIC = 390.5 µg mL^−1^)	*Penicillium* sp. (SCSGAF0023) isolated from *Dichotella gemmacea* collected in the South China Sea	[74]
	*B. megaterium* (zone of inhibition 15 mm)	*Blennoria* sp. isolated from *Carpobrotus edulis* collected at Gomera	[66]
Neocitreamicin I (**159**)	*B. subtilis* 1A1 (MIC = 0.06 μg mL^−1^); *S. aureus* (MRSA NRS1) (MIC = 0.50 μg mL^−1^); *S. aureus* (MRSA NRS2) (MIC = 0.12 μg mL^−1^); *S. aureus* (MRSA NRS71) (MIC = 0.12 μg mL^−1^); *E. faecalis* (VRE 51299) (MIC = 0.06 μg mL^−1^); *E. faecalis* (VRE 51575) (MIC = 0.12 μg mL^−1^); *E. coli* K-12 (MIC ≥ 8.0 μg mL^−1^)	*Nocardia* sp. (G0655) isolated from a sandy soil sample collected in Falmouth	[79]
Neocitreamicin II (**160**)	*B. subtilis* 1A1 (MIC = 0.12 μg mL^−1^); *S. aureus* (MRSA NRS1) (MIC = 1.0 μg mL^−1^); *S. aureus* (MRSA NRS2) (MIC = 0.50 μg mL^−1^); *S. aureus* (MRSA NRS71) (MIC = 0.50 μg mL^−1^); *E. faecalis* (VRE 51299) (MIC = 0.06 μg mL^−1^); *E. faecalis* (VRE 51575) (MIC = 0.25 μg mL^−1^); *E. coli* K-12 (MIC ≥ 8.0 μg mL^−1^)		
Citreamicin α (**161**)	*E. coli* (MIC > 128 μg mL^−1^); *K. pneumoniae* (MIC > 128 μg mL^−1^); *Serratia* sp. (MIC > 128 μg mL^−1^); *Citrobacter* sp. (MIC > 128 μg mL^−1^); *P. aeruginosa* (MIC ≥ 128 μg mL^−1^); *S. aureus* (MIC < 0.06~0.12 μg mL^−1^); *S. epidermidis* (MIC < 0.06 μg mL^−1^); *Enterococcus* sp. (MIC < 0.06~0.12 μg mL^−1^); *Streptococcus* sp. (MIC < 0.06 μg mL^−1^); *S. pneumoniae* (MIC < 0.06 μg mL^−1^); *B. fragilis* (MIC = 16 μg mL^−1^); *B. thetaiotaomicron* (MIC = 4 μg mL^−1^); *Clostridium perfringens* (MIC < 0.06 μg mL^−1^); *C. difficile* (MIC < 0.06 μg mL^−1^)	Culture LL-E19085 was isolated from a soil sample collected at Lake Manyara	[80]
Chrysoxanthone A (**162**)	*B. subtilis* (ATCC 63501) (MIC = 5 µg mL^−1^); *E. coli* (ATCC 25922) (MIC > 100 µg mL^−1^)	*Penicillium chrysogenum* (HLS111) isolated from a sponge	[50]
Chrysoxanthone B (**163**)	*S. epidermidis* (ATCC 12228, MSSE) (MIC = 10 µg mL^−1^); *S. aureus* (ATCC 29213, MSSA) (MIC = 20 µg mL^−1^); *B. subtilis* (ATCC 63501) (MIC = 5 µg mL^−1^); *E. faecalis* (ATCC 29212, VSE) (MIC ≥ 100 µg mL^−1^); *E. coli* (ATCC 25922) (MIC ≥ 100 µg mL^−1^)		
Chrysoxanthone C (**164**)	*S. epidermidis* (ATCC 12228, MSSE) (MIC = 20 µg mL^−1^); *S. aureus* (ATCC 29213, MSSA) (MIC = 80 µg mL^−1^); *B. subtilis* (ATCC 63501) (MIC = 10 µg mL^−1^); *E. faecalis* (ATCC 29212, VSE) (MIC > 100 µg mL^−1^); *E. coli* (ATCC 25922) (MIC > 100 µg mL^−1^)		
Ukixanthomycin A (**165**)	*B. cereus* (IC_50_ > 200 µM); *E. coli* (IC_50_ > 200 µM)	*Streptomyces* sp. (HGMA004) isolated from a mudflat collected at Uki	[58]

MIC: Minimum inhibitory concentration, IC_50_: Half maximal inhibitory concentration. *A. baumannii*: *Acinetobacter baumannii*; *A. hydrophila*: *Aeromonas hydrophila*; *B. anthracis*: *Bacillus anthracis*; *B. cereus*: *Bacillus cereus*; *B. fragilis*: *Bacteroides fragilis*; *B. megaterium*: *Bacillus megaterium*; *B. subtilis*: *Bacillus subtilis*; *B. thetaiotaomicron*: *Bacteroides thetaiotaomicron*; *C. difficile*: *Clostridium difficile*; *C. perfringens*: *Clostridium perfringens*; *E. coli*: *Escherichia coli*; *E. aerogenes*: *Enterobacter aerogenes*; *E. faecalis*: *Enterococcus faecalis*; *E. faecium*: *Enterococcus faecium*; *K. pneumoniae*: *Klebsiella pneumoniae*; *K. rhizophila*: *Kocuria rhizophila*; *M. bovis*: *Mycobacterium bovis*; *M. luteus*: *Micrococcus luteus*; *M. tuberculosis*: *Mycobacterium tuberculosis*; MRSA: Methicillin-resistant *Staphylococcus aureus*; *P. acnes*: *Propionibacterium acnes*; *P. aeruginosa*: *Pseudomonas aeruginosa*; *P. hauseri*: *Proteus hauseri*; *P. nigrifaciens*: *Pseudoalteromonas nigrifaciens*; *S. aureus*: *Staphyloccocus aureus*; *S. enterica*: *Salmonella enterica*; *S. epidermidis*: *Staphylococcus epidermidis*; *S. haemolyticus*: *Staphylococcus haemolyticus*; *S. pneumoniae*: *Streptococcus pneumoniae*; *S. saprophyticus*: *Staphylococcus saprophyticus*; *S. typhi*: *Salmonella typhi*; *S. ventriculi*: *Sarcina ventriculi*; *V. alginolyticus*: *Vibro alginolyticus*; *V. harveyi*: *Vibro harveyi*; *V. parahaemolyticus*: *Vibro parahaemolyticus*; *V. rotiferianus*: *Vibro rotiferianus*; *V. vulnificus*: *Vibro vulnificus*.

**Table 3:** Antifungal marine xanthone.

Name	Activity	Source	Ref.
Norlichexanthone (**17**)	*B. megaterium* (zone of inhibition 1 mm)	*Enteromorpha* sp. collected at Fehmarn Island	[81]
	*C. albicans* (ATCC 10231) (MIC = 6.25 μg mL^−1^); *A. niger* (ATCC 13496) (MIC = 25.0 μg mL^−1^); *F. oxysporum* f. sp. *cubense* (MIC = 50.0 μg mL^−1^)	*Talaromyces* sp. (ZH-154) collected in the South China Sea	[60]
Yicathin C (**18**)	*C. lagenarium* (zone of inhibition 11.0 mm)	*Aspergillus wentii* isolated from *Gymnogongrus flabelliformis* collected at Pingtan Island	[61]
Fischexanthone (**20**)	*F. graminearum* (MIC = 474.68 µM); *C. musae* (MIC = 474.68 µM)	*Alternaria* sp. (R6) isolated from mangrove collected at Leizhou peninsula	[62]
2,3,6,8-Tetrahydroxy-1-methylxanthone (**28**)	*M. violaceum* (zone of inhibition 1 mm)	*Enteromorpha* sp. collected at Fehmarn Island	[81]
Dimethyl 8-methoxy-9-oxo- 9H-xanthene-1, 6-dicarboxylate (**29**)	*F. oxysporum* f. sp. *cubense* (MIC = 12.5 µg mL^−1^)	*Penicillium* sp. ZZF 32# collected in the South China Sea	[82,83]
1-Hydroxy-6-methyl-8-(hydroxymethyl)xanthone (**30**)	*E. repens* (zone of inhibition 2 mm) *U. violacea* (zone of inhibition 2 mm)	*Ulocladium botrytis* (193A4) isolated from the *Callyspongia vaginalis* collected at Dominica	[84]
4-Chlorofischexanthone (**31**)	*F. graminearum* (MIC = 107 µM) *C. musae* (MIC = 214 µM)	*Alternaria* sp. (R6) isolated from mangrove collected at Leizhou peninsula	[62]
8-Hydroxy-3-methyl-9-oxo-9H-xanthene-1-carboxylic acid methyl ether (**32**)	*G. musae* (Rate of inhibition 53%); *P. cichoralearum* (Rate of inhibition 48%); *C. glocosporioides* (Rate of inhibition 28%); *B. graminearum* (Rate of inhibition 4.6%); *F. oxysporum* (Rate of inhibition 9.5%)	Co-culture broth of mangrove fungi (strain No. K38 and E33) collected in the South China Sea	[85,86]
Globosuxanthone A (**56**)	*C. albicans* IFM 4954 (zone of inhibition 7 mm)	*Beauveria bassiana* (TPU942) isolated from a sponge collected at Iriomote Island	[35]
Blennolide A (**65**)	*M. violaceum* (zone of inhibition 9 mm)	*Blennoria* sp. isolated from *Carpobrotus edulis* collected at Gomera	[66]
Blennolide B (**66**)	*M. violaceum* (zone of inhibition 8 mm)		
Paeciloxanthone (**68**)	*C. lunata* (zone of inhibition 6 mm); *C. albicans* (zone of inhibition 10 mm)	*Paecilomyces* sp. isolated from a mangrove collected in the Taiwan Strait	[40]
Versicone A (**74**)	C. a*cutatum* (MIC = 32 μg mL^−1^); *F. oxysporum* (MIC = 128 μg mL^−1^); *M. oryzae* (MIC > 200 μg mL^−1^)	*Aspergillus versicolor* (SCSIO 05879) collected in the Indian Ocean	[87]
Versicone B (**75**)	*C. acutatum* (MIC > 200 μg mL^−1^); *F. oxysporum* (MIC > 200 μg mL^−1^); *M. oryzae* (MIC > 200 μg mL^−1^)		
Versicone C (**76**)	*C.* a*cutatum* (MIC > 200 μg mL^−1^); *F. oxysporum* (MIC > 200 μg mL^−1^); *M. oryzae* (MIC > 200 μg mL^−1^)		
Versicone D (**77**)	*C. acutatum* (MIC > 200 μg mL^−1^); *F. oxysporum* (MIC > 200 μg mL^−1^); *M. oryzae* (MIC > 200 μg mL^−1^)		
Emerixanthone D (**109**)	*Fusarium* sp., *Penicillium* sp., *A. niger*, *R. solani*, *F. oxysporium* f. sp. *niveum*, *F. oxysporum* f. sp. *cucumeris*: Diameters of inhibition zones of which were both 3–4 mm	*Emericella* sp. (SCSIO 05240) collected in the South China Sea	[69]
Buanmycin (**156**)	*C. albicans* (MIC = 21.1 μM); *A. fumigatus* (MIC = 84.3 μM)	*Streptomyces* sp. isolated from a tidal mudflat collected in Buan	[57]
	*C. albicans* (IC_50_ = 0.4 μM)	*Streptomyces* sp. (HGMA004) isolated from a mudflat collected at Uki	[58]
Secalonic acid A (**157**)	*C. albicans* (ATCC 10231) (MIC = 6.25 μg mL^−1^); *A. niger* (ATCC 13496) (MIC = 6.25 μg mL^−1^); *F. oxysporum* f. sp. *cubense* (MIC = 12.5 μg mL^−1^)	*Talaromyces* sp. (ZH-154) collected in the South China Sea	[60]
Secalonic acid B (**158**)	*M. violaceum* (zone of inhibition 13 mm)	*Blennoria* sp. isolated from *Carpobrotus edulis* collected at Gomera	[66]
Ukixanthomycin A (**165**)	*C. albicans* (IC_50_ = 11.5 µM)	*Streptomyces* sp. (HGMA004) isolated from a mudflat collected at Uki	[58]

MIC: Minimum inhibitory concentration, IC_50_: Half maximal inhibitory concentration. *A. fumigatus*: *Aspergillus fumigatus*; *A. niger*: *Aspergillus niger*; *C. albicans*: *Candida albicans*; *C. acutatum*: *Colletotrichum acutatum*; *C. glocosporioides*: *Colletotrichum glocosporioides*; *C. lagenarium*: *Colletotrichum lagenarium*; *C. lunata*: *Curvularia lunata*; *C. musae*: *Calletotrichum musae*; *E. repens*: *Eurotium repens*; *F. graminearum*: *Fusarium graminearum*; *F. oxysporum*: *Fusarium oxysporum*; *G. musae*: *Gloeosporium musae*; *M. oryzae*: *Magnaporthe oryzae*; *M. violaceum*: *Microbotryum violaceum*; *P. cichoralearum*: *Peronophthora cichoralearum*; *R. solani*: *Rhizoctonia solani*; *U. violacea*: *Ustilago violacea*.

**Table 4:** Antiviral marine xanthone.

Name	Activity	Source	Ref.
Norlichexanthone (**17**)	EV71 (IC_50_ = 40.3 μM)	*Stachybotry* sp. (ZSDS1F1-2) isolated from a sponge collected at Xisha Island	[34]
	HIV-1-RT (82.9% inhibition at 66 μg mL^−1^)	*Enteromorpha* sp. collected at Fehmarn Island	[81]
2,3,6,8-Tetrahydroxy-1-methylxanthone (**28**)	HIV-1-RT (82.2% inhibition at 66 μg mL^−1^)		
3,8-Dihydroxy-6-methyl-9- oxo-9H-xanthene-1-carboxylate (**33**)	H1N1 (A/Puerto Rico/8/34 H274Y) (IC_50_ = 9.40 ± 1.96 µM); H1N1 (A/FM-1/1/47) (IC_50_ = 4.80 ± 1.28 µM); H3N2 (A/Aichi/2/68) (IC_50_ = 5.12 ± 1.49 µM)	*Diaporthe* sp. (SCSIO 41011), isolated from *Rhizophora stylosa*	[88]
Methyl-(2-chloro-l,6-dihydroxy-3-methylxanthone)-8-carboxylate (**34**)	H1N1 (IC_50_ = 133.4 µM); HSV-1 (IC_50_ = 55.5 µM); HSV-2 (IC_50_ = 175.5 µM)	*Aspergillus iizukae* collected from coastal saline soil	[89]
Methyl-(4-chloro-l,6-dihydroxy-3-methylxanthone)-8-carboxylate (**35**)	H1N1 (IC_50_ = 44.6 µM); HSV-1 (IC_50_ = 21.4 µM); HSV-2 (IC_50_ = 76.7 µM)		
Methyl-(4-chloro-6-hydroxy-1-methoxy-3-methylxanthone)-8-carboxylate **(36**)	H1N1 (IC_50_ ≥ 200 µM); HSV-1 (IC_50_ = 139.4 µM); HSV-2 (IC_50_ ≥ 200 µM)		
Methyl-(6-hydroxy-1-methoxy-3-methylxanthone)-8-carboxylate (**37**)	H1N1 (IC_50_ ≥ 200 µM); HSV-1 (IC_50_ = 157.7 µM); HSV-2 (IC_50_ = 163.3 µM)		
4-Chloro-1,6-dihydroxy-3-methylxanthone-8-carboxylic acid (**38**)	H1N1 (IC_50_ ≥ 200 µM); HSV-1 (IC_50_ = 183.3 µM); HSV-2 (IC_50_ ≥ 200 µM)		
2,4-Dichloro-1,6-dihydroxy-3-methylxanthone-8-carboxylic acid (**39**)	H1N1 (IC_50_ ≥ 200 µM); HSV-1 (IC_50_ = 144.4 µM); HSV-2 (IC_50_ ≥ 200 µM)		
Methyl-(l,6-dihydroxy-3-methylxanthone)-8-carboxylate (**40**)	H1N1 (IC_50_ = 140.4 µM); HSV-1 (IC_50_ = 75.7 µM); HSV-2 (IC_50_ = 95.4 µM)		
2-Hydroxy-1-(hydroxymethyl)-8-methoxy-3-methyl-9H-xanthen-9- one (**41**)	H1N1 (A/PuertoRico/8/34) (IC_50_ = 4.70 ± 1.11 µM); H1N1 (A/FM-1/1/47) (IC_50_ = 4.04 ± 0.58 µM)	*Aspergillus sydowii* (SCSIO 41.301) isolated from *Phakellia fusca*	[90]
2-Hydroxy-1-(hydroxymethyl)-7,8-dimethoxy-3-methyl-9H- xanthen-9-one (**42**)	H1N1 (A/PuertoRico/8/34) (IC_50_ = 2.17 ± 1.39 µM)		
Sterigmatocystin A (**110**)	HSV-2 (IC_50_ = 47.11 µM)	*Aspergillus versicolor* (15XS43ZD-1) strain was isolated from sponge collected from Xisha Islands, China	[91]
Sterigmatocystin B (**111**)	HSV-2 (IC_50_ = 39.45 µM)		
Sterigmatocystin C (**112**)	HSV-2 (IC_50_ = 38.73 µM)		
Asperxanthone (**113**)	Tobacco mosaic virus: inhibitory rate 62.9%	*Aspergillus* sp. collected in Quan-Zhou Gulf	[92]
Epiremisporine B (**121**)	EV71 (IC_50_ = 19.8 μM); H3N2 (IC_50_ = 24.1 μM)	*Penicillium* sp. (SCSIO Ind16F01) isolated from sediment collected in the Indian Ocean	[47]
Penicillixanthone A (**154**)	HIV-1 (SF162) (10 μM, 90.86 ± 0.82%); HIV-1 (CCR5-tropic) (IC_50_ = 0.36 µM); HIV-1 (CXCR4-tropic) (IC_50_ = 0.26 µM)	*Aspergillus fumigates* isolated from a jellyfish	[93]

EV71: Enterovirus 71; H1N1: Influenza A virus subtype H1N1; H3N2: Influenza A virus subtype H3N2; HIV: human immunodeficiency virus; HSV: herpes simplex virus; IC_50_: Half maximal inhibitory concentration; RT: Reverse-transcriptase.

**Table 5:** Antidiabetic marine xanthone.

Name	Activity	Source	Ref.
Chrysoxanthone (**48**)	α-Glucosidase inhibition (IC_50_ = 0.04 mM)	*Penicillium chrysogenum* (SCSIO 41001) isolated from sediment collected in the Indian Ocean	[94]
Staprexanthone A (**69**)	Pancreatic β-cell number (zebrafish model): ~40 at 10 µM	*Stachybotrys chartarum* (HDN16-358) isolated from mangrove collected in Fujian Province	[95]
Staprexanthone B (**70**)	Pancreatic β-cell number (zebrafish model): 40 at 10 µM		
Staprexanthone C (**71**)	Pancreatic β-cell number (zebrafish model): ~35 at 10 µM		
Staprexanthone D (**72**)	Pancreatic β-cell number (zebrafish model): ~35 at 10 µM		
Staprexanthone E (**73**)	Pancreatic β-cell number (zebrafish model): ~40 at 10 µM		
Austocystin J (**96**)	Inhibitory effect against phosphatases: SHP1 (IC_50_ = 15 μM); MEG2 (IC_50_ = 77 μM)	*Aspergillus puniceus* (SCSIO z021)	[96]
Austocystin K (**97**)	Inhibitory effect against phosphatases: TCPTP (IC_50_ = 16 μM); SHP1 (IC_50_ = 3.8 μM)		
Austocystin L (**98**)	Inhibitory effect against phosphatases: TCPTP (IC_50_ = 12 μM); SHP1 (IC_50_ = 20 μM); CDC25B (IC_50_ = 24 μM)		
Austocystin M (**99**)	Inhibitory effect against phosphatases: TCPTP (IC_50_ = 12 μM); SHP2 (IC_50_ = 9.5 μM); PTP1B (IC_50_ = 4.6 μM)		
Austocystin N (**100**)	Inhibitory effect against phosphatases: SHP1 (IC_50_ = 17 μM)		
Austocystin I (**101**)	Inhibitory effect against phosphatases: MEG2 (IC_50_ = 16 μM); CDC25B (IC_50_ = 19 μM)		
Austocystin F (**102**)	Inhibitory effect against phosphatases: SHP1 (IC_50_ = 6.7 μM); MEG2 (IC_50_ = 2.1 μM); CDC25B (IC_50_ = 6.7 μM); CD45 (IC_50_ = 20 μM)		
Austocystin A (**103**)	Inhibitory effect against phosphatases: TCPTP (IC_50_ = 19 μM); MEG2 (IC_50_ = 8.1 μM); CDC25B (IC_50_ = 16 μM)		
Austocystin H (**104**)	Inhibitory effect against phosphatases: TCPTP (IC_50_ = 3.0 μM); SHP1 (IC_50_ = 1.3 μM); SHP2 (IC_50_ = 1.3 μM); MEG2 (IC_50_ = 0.60 μM); PTP1B (IC_50_ = 0.90 μM); CDC25B (IC_50_ = 1.3 μM); CD45 (IC_50_ = 14 μM)		
Austocystin B (**105**)	Inhibitory effect against phosphatases: TCPTP (IC_50_ = 8.8 μM); SHP2 (IC_50_ = 2.0 μM); MEG2 (IC_50_ = 1.3 μM); PTP1B (IC_50_ = 1.8 μM); CDC25B (IC_50_ = 1.3 μM)		
Austocystin D (**106**)	Inhibitory effect against phosphatases: PTP1B (IC_50_ = 1.7 μM)		
8-*O*-Methyldihydrodemethylsterigmatocystin (**107**)	Inhibitory effect against phosphatases: TCPTP (IC_50_ = 11 μM); SHP1 (IC_50_ = 5.5 μM); MEG2 (IC_50_ = 4.6 μM); CDC25B (IC_50_ = 4.9 μM); CD45 (IC_50_ = 6.1 μM)		
(1′ R,2′ R)-compound V (**108**)	Inhibitory effect against phosphatases: TCPTP (IC_50_ = 19 μM); SHP1 (IC_50_ = 6.9 μM); MEG2 (IC_50_ = 4.2 μM)		

IC_50_: Half maximal inhibitory concentration.

**Table 6:** Antioxidant marine xanthone.

Name	Activity	Source	Ref.
1,4,7-Trihydroxy-6-methylxanthone (**15**)	DPPH (IC_50_ = 6.92 µg mL^−1^); ABTS (IC_50_ = 2.35 µg mL^−1^)	*Talaromyces islandicus* (EN-501) isolated from *Laurencia okamurai*	[59]
1,4,5-Trihydroxy-2-methylxanthone (**16**)	DPPH (IC_50_ = 1.23 µg mL^−1^); ABTS (IC_50_ = 1.27 µg mL^−1^)		
Norlichexanthone (**17**)	DPPH (% Scavenging effect: 6.2% at 25.0 μg mL^−1^; 12.9% at 50 μg mL^−1^; 25.3% at 100 μg mL^−1^; 90.6% at 500 μg mL^−1^)	*Enteromorpha* sp. collected at Fehmarn Island	[81]
2,3,6,8-Tetrahydroxy-1-methylxanthone (**28**)	DPPH (% Scavenging effect: 94.7% at 25.0 μg mL^−1^; 94.8% at 50 μg mL^−1^; 95.2% at 100 μg mL^−1^; 95.4% at 500 μg mL^−1^) Linolenic acid peroxidation (% Inhibition: 17.0% at 7.4 μg mL^−1^; 37.0% at 37 μg mL^−1^)		
Arthone C (**43**)	DPPH (IC_50_ = 16.9 µM); ABTS (IC_50_ = 18.7 µM)	*Arthrinium* sp. (UJNMF0008)	[97]
2,3,4,6,8-Pentahydroxy-1-methylxanthone (**44**)	DPPH (IC_50_ = 22.1 µM); ABTS (IC_50_ = 18.0 µM)		
Sterigmatocystin (**81**)	ABTS (0.65 ± 0.13 TEAC values)	*Aspergillus versicolor* (A-21-2-7) isolated from sediment collected in the South China Sea	[98]
Oxisterigmatocystin C (**93**)	ABTS (1.16 ± 0.18 TEAC values)		
Oxisterigmatocystin D (**120**)	ABTS (0.55 ± 0.13 TEAC values)		

ABTS: (2,2′-azino-bis(3-ethylbenzothiazoline-6-sulfonic acid)); DPPH: (2,2-diphenyl-1-picryl-hydrazyl-hydrate); IC_50_: Half maximal inhibitory concentration; TEAC: Trolox equivalents antioxidant capacity.

**Table 7:** Anti-inflammatory marine xanthone.

Name	Activity	Source	Ref.
Norlichexanthone (**17**)	COX-2 (IC_50_ = 34.3 µM)	*Stachybotry* sp. (ZSDS1F1-2) isolated from a sponge collected at Xisha Island	[34]
Yicathin C (**18**)	NO inhibition (27.0 ± 3.2%); NF-κB inhibition (56.8 ± 5.7%)	*Aspergillus europaeus* (WZXY-SX-4-1) isolated from *Xestospongia testudinaria*	[99]
	IL-6 cytokine % at 1 µM: 78.37 ± 7.78%	*Aspergillus wentii* isolated from *Gymnogongrus flabelliformis* collected at Pingtan Island	[4]
Yicathin B (**19**)	IL-6 cytokine % at 10 µM: 95.65 ± 17.21%		
	NO inhibition (35.3 ± 3.9%); NF-κB inhibition (81.2 ± 8.3%)	*Aspergillus europaeus* (WZXY-SX-4-1) isolated from *Xestospongia testudinaria*	[99]
1,3,6-trihydroxy-8-methylxanthone (**45**)	COX-2 (IC_50_ = 12.2 µM)	*Arthrinium* sp. (ZSDS1-F3) isolated from a sponge collected at Xisha Islands	[100]
Calyxanthone (**46**)	NO inhibition (17.6 ± 5.1) NF-κB: 63.7 ± 5.6	*Aspergillus europaeus* (WZXY-SX-4-1) isolated from *Xestospongia testudinaria*	[99]
Yicathin A (**47**)	NO inhibition (23.7 ± 4.8%); NF-κB inhibition (13.0 ± 9.8%)		
Euroanthone A (**166**)	NO inhibition (42.2 ± 2.3%); NF-κB inhibition (68.8 ± 7.0%)		
Euroanthone B (**167**)	NO inhibition (23.4 ± 3.3%); NF-κB inhibition (52.3 ± 10.6%)		

COX: Cyclooxygenase; IC_50_: Half maximal inhibitory concentration; NF-κΒ: factor nuclear kappa B; NO: nitric oxide.

**Table 8:** Marine xanthone with miscellaneous biological activities.

Name	Activity	Source	Ref.
Sydowinin A (**2**)	Immunosuppressive: Inhibition of Con A-Induced proliferation (IC_50_ = 6.5 μg mL^−1^); Inhibition of LPS-Induced proliferation (IC_50_ = 7.1 μg mL^−1^)	*Penicillium* sp. (ZJ-SY2) isolated from *Sonneratia apetala*	[101]
Sydowinin B (**3**)	Immunosuppressive: Inhibition of Con A-Induced proliferation (IC_50_ = 19.2 μg mL^−1^); Inhibition of LPS-Induced proliferation (IC_50_ = 20.8 μg mL^−1^)		
Methyl 8-hydroxy-6-methyl-9-oxo-9H-xanthene-1- carboxylate (**7**)	Immunosuppressive: Inhibition of Con A-Induced proliferation (IC_50_ = 25.7 μg mL^−1^); Inhibition of LPS-Induced proliferation (IC_50_ = 26.4 μg mL^−1^)		
Conioxanthone A (**12**)	Immunosuppressive: Inhibition of Con A-Induced proliferation (IC_50_ = 8.2 μg mL^−1^); Inhibition of LPS-Induced proliferation (IC_50_ = 7.5 μg mL^−1^)		
Pinselin (**49**)	Immunosuppressive: Inhibition of Con A-Induced proliferation (IC_50_ = 5.9 μg mL^−1^); Inhibition of LPS-Induced proliferation (IC_50_ = 7.5 μg mL^−1^)		
Epiremisporine B (**121**)	Immunosuppressive: Inhibition of Con A-Induced proliferation (IC_50_ = 30.8 μg mL^−1^); Inhibition of LPS-Induced proliferation (IC_50_ = 31.2 μg mL^−1^)		
Remisporine B (**169**)	Immunosuppressive: Inhibition of Con A-Induced proliferation (IC_50_ = 30.1 μg mL^−1^); Inhibition of LPS-Induced proliferation (IC_50_ = 32.4 μg mL^−1^)		
Paeciloxanthone (**68**)	Anti-Alzheimer: acetylcholinesterase inhibition (IC_50_ = 2.25 µg mL^−1^)	*Paecilomyces* sp. isolated from a mangrove collected in the Taiwan Strait	[40]
Chaetoxanthone A (**78**)	Antiprotozoal: *T. brucei rhodesiense* (strain STIB 900) (IC_50_ = 4.7 μg mL^−1^); *T. cruzi* (strain Tulahuen C4) (IC_50_ ≥ 10 μg mL^−1^); *L. donovani* (strain MHOM-ET-67/L82) (IC_50_ = 5.3 μg mL^−1^); *P. falciparum* (IC_50_ 3.5 μg mL^−1^)	*Chaetomium* sp. isolated from the Greek alga collected at Santorini Island	[41,102,103]
Chaetoxanthone B (**79**)	Antiprotozoal: *T. brucei rhodesiense* (strain STIB 900) (IC_50_ = 9.3 μg mL^−1^); *T. cruzi* (strain Tulahuen C4) (IC_50_ = 7.1 μg mL^−1^); *L. donovani* (strain MHOM-ET-67/L82) (IC_50_ = 3.4 μg mL^−1^); *P. falciparum* (IC_50_ = 0.5 μg mL^−1^)		
Chaetoxanthone C (**80**)	Antiprotozoal: *T. brucei rhodesiense* (strain STIB 900) (IC_50_ = 42.6 μg mL^−1^); *T. cruzi* (strain Tulahuen C4) (IC_50_ = 1.5 μg mL^−1^); *L. donovani* (strain MHOM-ET-67/L82) (IC_50_ = 3.1 μg mL^−1^); *P. falciparum* (IC_50_ = 4.0 μg mL^−1^)		
Aspergixanthone A (**82**)	Against aquatic pathogens: *V. parahemolyticus* (MIC = 25.0 µM); *V. anguillarum* (MIC = 25.0 µM); *V. alginolyticus* (MIC = 25.0 µM)	*Aspergillus* sp. (ZA-01)	[104]
Sterigmatocystin A (**110**)	Angiogenesis: Increase length of intersomitic vessels of transgenic zebrafish at 1.25 μM	*Aspergillus versicolor* (15XS43ZD-1) isolated from a sponge collected at Xisha Island	[91]
Aspergixanthone I (**114**)	Against aquatic pathogens: *V. parahemolyticus* (MIC = 1.56 µM); *V. anguillarum* (MIC = 1.56 µM); *V. alginolyticus* (MIC = 3.12 µM)	*Aspergillus* sp. (ZA-01)	[104]
Aspergixanthone J (**115**)	Against aquatic pathogens: *V. parahemolyticus* (MIC = 6.25µM); *V. anguillarum* (MIC = 25.0 µM); *V. alginolyticus* (MIC = 25.0 µM)		
Aspergixanthone K (**116**)	Against aquatic pathogens: *V. parahemolyticus* (MIC = 3.12 µM); *V. anguillarum* (MIC = 25.0 µM); *V. alginolyticus* (MIC = 12.5 µM)		
15-Acetyl tajixanthone hydrate (**117**)	Against aquatic pathogens: *V. parahemolyticus* (MIC = 12.5 µM); *V. anguillarum* (MIC = 25.0 µM); *V. alginolyticus* (MIC = 12.5 µM)		
Tajixanthone hydrate (**118**)	Against aquatic pathogens: *V. parahemolyticus* (MIC = 6.25 µM); *V. anguillarum* (MIC = 6.25 µM); *V. alginolyticus* (MIC = 12.5 µM)		
16-Chlorotajixanthone (**119**)	Against aquatic pathogens: *V. parahemolyticus* (MIC = 25.0 µM); *V. anguillarum* (MIC = 6.25 µM); *V. alginolyticus* (MIC = 25.0 µM)		
Isosecosterigmatocystin (**169**)	Against aquatic pathogens: *Ed. ictaluri* (IC_50_ = 16 μg mL^−1^)	*Aspergillus nidulans* (MA-143) isolated from *Rhizophora stylosa*	[105]

Con A: Concanavalin A; *Ed. ictaluri*: *Edwardsiella ictaluri*; IC_50_: Half maximal inhibitory concentration; MIC: Minimum inhibitory concentration, *L. donovani*: *Leishmania donovani*; LPS: Lipopolysaccharide; *T. brucei*: *Trypanosoma brucei*; *T. cruzi*: *Trypanosoma cruzi*; *V. alginolyticus*: *Vibrio alginolyticus*; *V. anguillarum*: *Vibrio anguillarum*; *V. parahemolyticus*: *Vibrio parahemolyticus*.

## Data Availability

The data presented in this study are available in the article.

## Supplementary Materials

The following are available online at https://www.mdpi.com/article/10.3390/md20010058/s1, Table S1: Molecular descriptors of the bioactive marine xanthones, Table S2: NP-likeness and drug-likeness scores of the bioactive marine xanthones, Table S3: Chemical functional groups present in bioactive marine xanthones.
