# Supplementary material for: Bioactive Marine Xanthones: A Review
Source: Mar Drugs. 2022 Jan 8;20(1):58. doi: 10.3390/md20010058 (PMC8778107; doi:10.3390/md20010058)
Supplement: Supplementary file 1 [file marinedrugs-20-00058-s001.zip › marinedrugs-1537547-supplementary.pdf]

marine drugs

MDPI

Supplementary Materials

Bioactive Marine Xanthones: A Review

José X. Soares [1,2,†], Daniela R. P. Loureiro [1,2,3,†], Ana Laura Dias [2], Salete Reis [1], Madalena M. M. Pinto [2,3] and Carlos M. M. Afonso [2,3,*]

[1] Laboratório Associado para a Química Verde (LAQV), REQUIMTE (Rede de Química e Tecnologia), Department of Chemical Sciences, Faculty of Pharmacy, University of Porto, Rua de Jorge Viterbo Ferreira, 228, 4050-313 Porto, Portugal; jfxsoares@ff.up.pt (J.X.S); dloureiro@ff.up.pt (D.R.P.L.); shreis@ff.up.pt (S.R.)
[2] Laboratory of Organic and Pharmaceutical Chemistry, Department of Chemical Sciences, Faculty of Pharmacy, University of Porto, Rua de Jorge Viterbo Ferreira, 228, 4050-313 Porto, Portugal; dloureiro@ff.up.pt (D.R.P.L.); up201903848@ff.up.pt (A.L.D.); madalena@ff.up.pt (M.M.M.P.); cafonso@ff.up.pt (C.M.M.A.)
3 Interdisciplinary Center of Marine and Environmental Investigation (CIIMAR/CIMAR), Edifício do Terminal de Cruzeiros do Porto de Leixões, Av. General Norton de Matos s/n, 4050-208 Matosinhos, Portugal; dloureiro@ff.up.pt (D.R.P.L.); madalena@ff.up.pt (M.M.M.P.); cafonso@ff.up.pt (C.M.M.A.)
* Correspondence: cafonso@ff.up.pt; Tel.: +351-22-042-8500
† These authors contributed equally to this work.

Table of Content

Table S1 Molecular descriptors of the bioactive marine xanthones 1
Table S2 NP-likeness and drug-likeness scores of the bioactive marine xanthones 6
Table S3 Chemical functional groups present in bioactive marine xanthones. 10

Table S1 Molecular descriptors of the bioactive marine xanthones

Compound	MW	Fsp3	Number of RB	TPSA	Log S	Log P
**1**	334.71	0.13	2	96.97	-3.90	2.58
**2**	300.27	0.13	2	96.97	-3.32	1.93
**3**	316.27	0.13	2	117.20	-3.51	1.64
**4**	300.27	0.13	1	96.97	-3.60	2.45
**5**	270.24	0.07	1	87.74	-3.45	2.66
**6**	270.24	0.07	1	87.74	-3.89	2.66
**7**	284.27	0.13	1	76.74	-4.09	2.75
**8**	300.27	0.13	2	96.97	-3.32	1.93
**9**	270.24	0.07	1	76.74	-3.80	2.44
**10**	314.25	0.06	2	114.04	-3.64	2.14
**11**	300.27	0.13	1	96.97	-3.94	2.45
**12**	316.27	0.13	2	117.20	-3.17	1.64
**13**	272.26	0.13	1	79.90	-3.96	2.67
**14**	330.25	0.06	2	134.27	-3.49	1.84
**15**	258.23	0.07	0	90.90	-3.76	2.37
**16**	258.23	0.07	0	90.90	-3.76	2.37
**17**	258.23	0.07	0	90.90	-3.76	2.37
**18**	300.27	0.13	2	96.97	-3.94	2.67
**19**	314.29	0.18	2	85.97	-4.15	2.76
**20**	316.27	0.13	2	117.20	-3.61	1.64
**21**	334.71	0.13	1	96.97	-4.23	3.11

**22**	334.71	0.13	1	96.97	-4.23	3.11
**23**	314.25	0.06	2	114.04	-3.89	1.96
**24**	342.30	0.17	3	103.04	-3.96	2.50
**25**	258.23	0.07	0	90.90	-3.76	2.37
**26**	302.28	0.25	1	96.97	-3.99	1.45
**27**	349.34	0.05	3	102.93	-4.90	3.59
**28**	274.23	0.07	0	111.13	-3.61	2.08
**29**	342.30	0.17	3	92.04	-3.71	2.53
**30**	256.26	0.13	1	70.67	-3.57	2.45
**31**	350.71	0.13	2	117.20	-4.20	2.29
**32**	284.27	0.13	1	76.74	-4.09	2.75
**33**	300.27	0.13	1	96.97	-3.94	2.45
**34**	334.71	0.13	1	96.97	-4.53	3.11
**35**	334.71	0.13	1	96.97	-4.53	3.11
**36**	348.74	0.18	2	85.97	-4.39	3.41
**37**	314.29	0.18	2	85.97	-3.81	2.76
**38**	320.68	0.07	1	107.97	-4.32	3.02
**39**	355.13	0.07	1	107.97	-4.91	3.67
**40**	300.27	0.13	1	96.97	-3.94	2.45
**41**	286.28	0.19	2	79.90	-3.62	2.46
**42**	316.31	0.24	3	89.13	-3.68	2.47
**43**	332.26	0.13	2	137.43	-3.37	1.34
**44**	290.23	0.07	0	131.36	-3.46	1.78
**45**	258.23	0.07	0	90.90	-3.76	2.37
**46**	286.24	0.07	1	107.97	-3.74	2.36
**47**	314.29	0.18	2	85.97	-4.15	2.76
**48**	385.16	0.13	1	117.20	-5.33	3.46
**49**	300.27	0.13	1	96.97	-4.09	2.45
**50**	318.28	0.25	2	117.20	-2.54	0.64
**51**	320.30	0.38	2	117.20	-3.51	0.55
**52**	320.30	0.38	1	117.20	-2.74	0.34
**53**	320.30	0.38	2	117.20	-2.59	0.55
**54**	336.30	0.38	2	137.43	-2.78	-0.47
**55**	336.30	0.38	2	137.43	-2.07	-0.47
**56**	304.25	0.20	1	117.20	-2.61	0.25
**57**	294.30	0.53	0	107.22	-2.19	0.14
**58**	278.30	0.53	0	86.99	-2.76	1.10
**59**	308.33	0.56	1	96.22	-2.85	1.11
**60**	320.30	0.38	1	117.20	-2.88	0.47
**61**	320.30	0.38	1	117.20	-2.88	0.47
**62**	260.29	0.40	0	70.67	-3.45	2.16
**63**	336.30	0.38	2	137.43	-2.20	-0.34
**64**	318.28	0.25	2	117.20	-2.54	0.64
**65**	320.30	0.38	1	113.29	-3.18	1.09
**66**	320.30	0.38	1	113.29	-2.82	1.09

**67**	426.47	0.33	7	84.20	-5.08	4.68
**68**	324.38	0.25	3	70.67	-4.96	3.96
**69**	394.47	0.38	3	68.90	-6.05	5.29
**70**	392.50	0.32	5	70.67	-6.40	5.42
**71**	392.50	0.32	5	70.67	-6.39	5.47
**72**	392.50	0.32	5	70.67	-6.40	5.42
**73**	392.50	0.32	5	70.67	-6.40	5.42
**74**	354.40	0.29	5	68.90	-4.55	4.10
**75**	384.43	0.32	6	78.13	-4.62	4.11
**76**	370.40	0.29	5	89.13	-4.41	3.81
**77**	354.40	0.29	5	68.90	-4.69	4.45
**78**	370.36	0.35	1	98.36	-4.21	2.98
**79**	354.36	0.35	1	78.13	-4.73	4.01
**80**	390.82	0.35	2	89.13	-5.27	4.65
**81**	324.29	0.17	1	78.13	-4.34	3.01
**82**	512.56	0.43	6	135.66	-6.17	3.97
**83**	498.53	0.41	5	146.66	-5.82	3.31
**84**	512.56	0.43	6	135.66	-5.82	3.97
**85**	354.31	0.21	2	87.36	-4.41	3.02
**86**	342.30	0.28	1	98.36	-3.92	2.20
**87**	384.34	0.30	2	104.43	-4.37	2.77
**88**	356.33	0.32	2	87.36	-4.24	2.89
**89**	498.53	0.41	5	146.66	-4.70	3.31
**90**	458.94	0.40	4	100.13	-5.76	4.50
**91**	468.55	0.44	6	98.36	-4.75	4.56
**92**	468.50	0.38	6	126.43	-5.85	3.43
**93**	356.33	0.32	2	87.36	-4.26	2.86
**94**	480.51	0.33	5	126.43	-5.42	4.12
**95**	438.48	0.32	4	120.36	-4.96	3.55
**96**	370.31	0.21	2	107.59	-3.81	2.12
**97**	402.79	0.25	3	76.36	-4.87	3.97
**98**	408.41	0.26	3	98.36	-5.17	3.62
**99**	394.38	0.23	2	109.36	-4.96	3.32
**100**	398.41	0.41	3	109.36	-4.55	3.28
**101**	340.29	0.17	1	98.36	-3.74	2.11
**102**	326.26	0.12	0	109.36	-3.53	1.81
**103**	372.76	0.21	2	67.13	-4.20	3.96
**104**	394.38	0.23	2	109.36	-4.96	3.32
**105**	396.40	0.32	3	109.36	-4.72	3.41
**106**	412.39	0.32	3	129.59	-4.13	2.51
**107**	326.30	0.28	1	78.13	-4.16	2.88
**108**	360.71	0.12	0	109.36	-4.12	2.46
**109**	482.53	0.41	5	126.43	-4.51	3.82
**110**	356.33	0.32	2	87.36	-4.26	2.86
**111**	398.41	0.41	5	87.36	-5.07	4.03

**112**	398.41	0.41	5	87.36	-5.07	4.03
**113**	324.29	0.17	1	78.13	-4.60	3.01
**114**	482.53	0.41	5	126.43	-5.31	3.82
**115**	494.54	0.36	6	115.43	-5.77	4.77
**116**	452.50	0.35	5	109.36	-5.30	4.20
**117**	482.53	0.41	5	126.43	-5.31	3.82
**118**	440.49	0.40	4	120.36	-4.84	3.25
**119**	458.94	0.40	4	100.13	-5.76	4.50
**120**	356.33	0.32	2	87.36	-4.26	2.86
**121**	576.51	0.33	2	182.94	-5.28	2.12
**122**	750.71	0.42	7	238.72	-6.14	4.09
**123**	638.58	0.38	3	226.58	-5.05	2.16
**124**	638.58	0.38	3	226.58	-6.01	2.23
**125**	638.58	0.38	3	226.58	-6.01	2.23
**126**	636.56	0.25	5	237.58	-6.71	2.91
**127**	654.58	0.38	4	246.81	-5.32	1.14
**128**	654.58	0.38	4	246.81	-5.32	1.14
**129**	638.58	0.38	3	226.58	-6.06	2.16
**130**	638.58	0.38	3	226.58	-6.06	2.16
**131**	575.53	0.30	2	187.20	-6.25	2.57
**132**	575.53	0.30	2	187.20	-6.25	2.57
**133**	666.63	0.44	6	212.42	-7.42	3.07
**134**	612.63	0.23	10	130.73	-5.53	5.97
**135**	666.63	0.41	5	226.58	-5.15	2.94
**136**	666.63	0.41	5	226.58	-5.15	2.94
**137**	708.67	0.42	6	232.65	-5.62	3.52
**138**	666.63	0.41	5	226.58	-5.57	2.94
**139**	708.67	0.42	6	232.65	-6.06	3.52
**140**	638.58	0.38	3	226.58	-6.06	2.16
**141**	750.71	0.42	7	238.72	-5.92	4.09
**142**	616.58	0.33	2	197.12	-5.66	3.45
**143**	616.58	0.33	2	197.12	-5.66	3.45
**144**	616.58	0.33	2	197.12	-5.66	3.45
**145**	630.60	0.35	2	197.12	-5.93	3.84
**146**	630.60	0.35	2	197.12	-5.93	3.84
**147**	630.60	0.35	2	197.12	-5.93	3.84
**148**	630.60	0.35	2	197.12	-5.93	3.84
**149**	954.84	0.40	13	360.72	-5.30	-1.73
**150**	575.53	0.30	2	187.20	-6.25	2.57
**151**	575.53	0.30	2	187.20	-6.25	2.57
**152**	520.45	0.22	2	183.96	-6.30	3.31
**153**	504.45	0.22	2	163.73	-6.44	3.60
**154**	638.58	0.38	3	226.58	-6.44	2.16
**155**	690.65	0.39	7	175.49	-7.36	3.71
**156**	593.54	0.27	7	224.06	-4.96	2.24

**157**	638.58	0.38	3	226.58	-6.06	2.16
**158**	638.58	0.38	3	226.58	-6.06	2.16
**159**	653.64	0.33	5	177.72	-7.48	4.39
**160**	825.82	0.43	8	222.48	-8.38	4.49
**161**	669.64	0.33	6	175.95	-7.11	4.06
**162**	638.58	0.41	4	212.42	-5.84	2.29
**163**	638.58	0.41	4	212.42	-5.84	2.29
**164**	638.58	0.41	4	212.42	-5.84	2.29
**165**	561.50	0.21	4	199.89	-5.94	2.78
**166**	418.35	0.25	5	163.73	-3.31	0.96
**167**	404.33	0.21	5	174.73	-3.10	0.87
**168**	576.51	0.33	2	182.94	-5.28	2.12
**169**	360.36	0.32	5	120.36	-2.68	2.21

MW: molecular weight; Fsp^3: fraction of sp^3 carbons; RB: rotatable bonds; Log P: lipophilicity; TPSA: topological polar surface area; Log S: solubility

Table S2 NP-likeness and drug-likeness scores of the bioactive marine xanthones

Compound	NP-likeness score	QED score	Lipinski compliance	Ghose compliance	Veber compliance	Egan compliance	Gleeson compliance
**1**	0.73	0.55	1	1	1	1	0.5
**2**	1.05	0.55	1	1	1	1	0.5
**3**	1.27	0.49	1	1	1	1	0.5
**4**	1.08	0.53	1	1	1	1	0.5
**5**	1.20	0.58	1	1	1	1	0.5
**6**	0.93	0.66	1	1	1	1	0.5
**7**	0.84	0.55	1	1	1	1	0.5
**8**	1.05	0.55	1	1	1	1	0.5
**9**	0.73	0.54	1	1	1	1	0.5
**10**	0.93	0.55	1	1	1	1	0.5
**11**	1.15	0.53	1	1	1	1	0.5
**12**	1.34	0.49	1	1	1	1	0.5
**13**	1.22	0.67	1	1	1	1	0.5
**14**	1.03	0.48	1	1	1	0.5	0.5
**15**	1.25	0.43	1	1	1	1	0.5
**16**	1.32	0.43	1	1	1	1	0.5
**17**	1.43	0.54	1	1	1	1	0.5
**18**	1.03	0.71	1	1	1	1	0.5
**19**	0.93	0.58	1	1	1	1	0.5
**20**	1.03	0.56	1	1	1	1	0.5
**21**	0.81	0.53	1	1	1	1	0.5
**22**	0.89	0.53	1	1	1	1	0.5
**23**	1.10	0.55	1	1	1	1	0.5
**24**	1.05	0.55	1	1	1	1	0.5
**25**	1.44	0.54	1	1	1	1	0.5
**26**	1.28	0.39	1	1	0.5	0.5	0.5
**27**	0.59	0.33	1	1	1	1	0.5
**28**	1.74	0.37	1	1	1	1	0.5
**29**	0.69	0.53	1	1	1	1	0.5
**30**	0.96	0.66	1	1	1	1	0.5
**31**	0.93	0.55	1	1	1	1	0.5
**32**	0.73	0.55	1	1	1	1	0.5
**33**	1.15	0.53	1	1	1	1	0.5
**34**	1.05	0.52	1	1	1	1	0.5
**35**	1.01	0.52	1	1	1	1	0.5
**36**	0.83	0.56	1	1	1	1	0.5
**37**	0.96	0.58	1	1	1	1	0.5
**38**	1.12	0.59	1	1	1	1	0.5
**39**	1.06	0.58	1	1	1	1	0.5
**40**	1.15	0.53	1	1	1	1	0.5
**41**	1.07	0.71	1	1	1	1	0.5

**42**	1.08	0.72	1	1	1	1	0.5
**43**	1.64	0.31	1	1	1	0.5	0.5
**44**	1.98	0.31	1	1	1	1	0.5
**45**	1.43	0.54	1	1	1	1	0.5
**46**	1.27	0.59	1	1	1	1	0.5
**47**	0.85	0.58	1	1	1	1	0.5
**48**	1.28	0.33	1	1	1	1	0.5
**49**	0.84	0.55	1	1	1	1	0.5
**50**	1.45	0.70	1	1	1	1	0.5
**51**	1.27	0.49	1	1	1	1	0.5
**52**	2.25	0.63	1	1	1	1	0.5
**53**	1.70	0.69	1	1	1	1	0.5
**54**	1.40	0.82	1	1	1	1	0.5
**55**	1.99	0.54	1	0.75	1	0.5	0.5
**56**	1.43	0.65	1	1	1	1	0.5
**57**	2.35	0.54	1	1	1	1	0.5
**58**	1.94	0.66	1	1	1	1	0.5
**59**	1.95	0.72	1	1	1	1	0.5
**60**	1.88	0.64	1	1	1	1	0.5
**61**	1.88	0.64	1	1	1	1	0.5
**62**	2.28	0.75	1	1	1	1	0.5
**63**	1.88	0.55	1	0.75	1	0.5	0.5
**64**	1.45	0.70	1	1	1	1	0.5
**65**	1.59	0.66	1	1	1	1	0.5
**66**	1.74	0.66	1	1	1	1	0.5
**67**	1.01	0.31	1	1	1	1	0
**68**	1.19	0.56	1	1	1	1	0.5
**69**	1.51	0.48	1	1	1	1	0
**70**	1.48	0.45	1	1	1	1	0
**71**	1.42	0.45	1	1	1	1	0
**72**	1.49	0.45	1	1	1	1	0
**73**	1.34	0.45	1	1	1	1	0
**74**	1.06	0.55	1	1	1	1	0
**75**	1.07	0.51	1	1	1	1	0
**76**	1.22	0.52	1	1	1	1	0.5
**77**	1.19	0.56	1	1	1	1	0.5
**78**	1.80	0.64	1	1	1	1	0.5
**79**	1.58	0.67	1	1	1	1	0
**80**	1.64	0.62	1	1	1	1	0
**81**	1.76	0.69	1	1	1	1	0.5
**82**	1.72	0.27	0.8	0.25	1	1	0
**83**	1.77	0.27	1	0.5	1	1	0
**84**	1.68	0.27	1	0.5	1	1	0
**85**	1.70	0.71	1	1	1	1	0.5
**86**	2.03	0.65	1	1	1	1	0.5

**87**	1.80	0.53	1	1	1	1	0.5
**88**	1.54	0.71	1	1	1	1	0.5
**89**	1.99	0.24	1	0.5	0.5	0.5	0.5
**90**	1.77	0.30	1	1	1	1	0
**91**	1.61	0.53	1	1	1	1	0.5
**92**	1.85	0.33	1	1	1	1	0.5
**93**	1.68	0.71	1	1	1	1	0.5
**94**	1.93	0.28	1	0.5	1	1	0
**95**	1.98	0.36	1	1	1	1	0.5
**96**	1.58	0.66	1	1	1	1	0.5
**97**	1.38	0.61	1	1	1	1	0.5
**98**	1.65	0.50	1	1	1	1	0.5
**99**	1.80	0.45	1	1	1	1	0.5
**100**	1.92	0.58	1	1	1	1	0.5
**101**	1.62	0.65	1	1	1	1	0.5
**102**	1.84	0.54	1	1	1	1	0.5
**103**	1.29	0.67	1	1	1	1	0.5
**104**	1.81	0.45	1	1	1	1	0.5
**105**	2.07	0.58	1	1	1	1	0.5
**106**	1.96	0.48	1	1	1	1	0.5
**107**	1.61	0.69	1	1	1	1	0.5
**108**	1.60	0.53	1	1	1	1	0.5
**109**	1.75	0.37	1	0.75	0.5	0.5	0.5
**110**	1.68	0.71	1	1	1	1	0.5
**111**	1.69	0.51	1	1	1	1	0
**112**	1.69	0.51	1	1	1	1	0
**113**	1.71	0.69	1	1	1	1	0.5
**114**	1.89	0.28	1	0.5	1	1	0.5
**115**	1.86	0.29	1	0.5	1	1	0
**116**	1.91	0.39	1	1	1	1	0
**117**	1.89	0.28	1	0.5	1	1	0.5
**118**	1.94	0.36	1	1	1	1	0.5
**119**	1.77	0.30	1	1	1	1	0
**120**	1.68	0.71	1	1	1	1	0.5
**121**	1.59	0.38	0.6	0.5	0.5	0.5	0.5
**122**	1.80	0.24	0.6	0.25	0	0.5	0
**123**	1.63	0.26	0.4	0.25	0.5	0.5	0.5
**124**	1.82	0.27	0.4	0.25	0.5	0.5	0.5
**125**	1.82	0.27	0.4	0.25	0.5	0.5	0.5
**126**	1.52	0.12	0.4	0.25	0.5	0.5	0.5
**127**	1.82	0.23	0.4	0.25	0.5	0.5	0.5
**128**	1.82	0.23	0.4	0.25	0.5	0.5	0.5
**129**	1.74	0.27	0.4	0.25	0.5	0.5	0.5
**130**	1.74	0.27	0.4	0.25	0.5	0.5	0.5
**131**	1.45	0.13	0.6	0.5	0.5	0.5	0.5

**132**	1.45	0.13	0.6	0.5	0.5	0.5	0.5
**133**	0.83	0.15	0.8	0	1	0.5	0
**134**	1.81	0.30	0.6	0.25	0.5	0.5	0.5
**135**	2.02	0.25	0.4	0.25	0.5	0.5	0.5
**136**	2.00	0.25	0.4	0.25	0.5	0.5	0.5
**137**	1.94	0.21	0.6	0.25	0.5	0.5	0.5
**138**	2.07	0.30	0.6	0.25	0.5	0.5	0.5
**139**	1.99	0.26	0.6	0.25	0.5	0.5	0
**140**	1.74	0.27	0.4	0.25	0.5	0.5	0.5
**141**	1.86	0.23	0.6	0.25	0.5	0.5	0
**142**	1.87	0.24	0.6	0.25	0.5	0.5	0.5
**143**	1.87	0.24	0.6	0.25	0.5	0.5	0.5
**144**	1.89	0.24	0.6	0.25	0.5	0.5	0
**145**	1.88	0.24	0.6	0.25	0.5	0.5	0.5
**146**	1.78	0.24	0.6	0.25	0.5	0.5	0
**147**	1.89	0.24	0.6	0.25	0.5	0.5	0.5
**148**	1.89	0.24	0.6	0.25	0.5	0.5	0.5
**149**	1.55	0.05	0.4	0.25	0	0.5	0.5
**150**	1.44	0.13	0.6	0.5	0.5	0.5	0.5
**151**	1.44	0.13	0.6	0.5	0.5	0.5	0.5
**152**	1.62	0.15	0.6	0.5	0.5	0.5	0.5
**153**	1.48	0.23	0.8	0.5	0.5	0.5	0.5
**154**	1.72	0.27	0.4	0.25	0.5	0.5	0.5
**155**	1.52	0.25	0.6	0.25	0.5	0.5	0
**156**	1.46	0.12	0.4	0.5	0.5	0.5	0.5
**157**	1.72	0.27	0.4	0.25	0.5	0.5	0.5
**158**	1.72	0.27	0.4	0.25	0.5	0.5	0.5
**159**	1.15	0.26	0.6	0.25	0.5	0.5	0
**160**	1.24	0.16	0.6	0.25	0.5	0.5	0
**161**	1.05	0.26	0.6	0.25	0.5	0.5	0
**162**	1.76	0.28	0.6	0.25	0.5	0.5	0.5
**163**	1.76	0.28	0.6	0.25	0.5	0.5	0.5
**164**	1.76	0.28	0.6	0.25	0.5	0.5	0.5
**165**	1.29	0.18	1	0.75	0.5	0.5	0.5
**166**	1.42	0.35	1	1	0.5	0.5	0.5
**167**	1.53	0.30	1	1	0.5	0.5	0.5
**168**	1.59	0.38	0.6	0.5	0.5	0.5	0.5
**169**	1.98	0.42	1	1	0.5	0.5	0.5

Compliance with Lipinski, Ghose, Veber, Egan, and Gleeson was calculated with the following formula: (n° of criteria – n° of violations) / n° of criteria

marine drugs

MDPI

Table S3 Chemical functional groups present in bioactive marine xanthones.

Compound	Phenol	Ketone	Ester	COOH	Alcohol	Lactone	Aryl methyl	Methoxy	Halogen	Amine 2°	Amine 3°	Amide
**1**	1	0	1	0	1	0	0	1	1	0	0	0
**2**	1	0	1	0	1	0	0	1	0	0	0	0
**3**	2	0	1	0	1	0	0	1	0	0	0	0
**4**	2	0	1	0	0	0	1	1	0	0	0	0
**5**	1	0	0	1	0	0	1	0	0	0	0	0
**6**	1	0	0	1	0	0	1	0	0	0	0	0
**7**	1	0	1	0	0	0	1	1	0	0	0	0
**8**	1	0	1	0	1	0	0	1	0	0	0	0
**9**	1	0	1	0	0	0	0	1	0	0	0	0
**10**	1	0	1	1	0	0	0	1	0	0	0	0
**11**	2	0	1	0	0	0	1	1	0	0	0	0
**12**	2	0	1	0	1	0	0	1	0	0	0	0
**13**	2	0	0	0	0	0	1	1	0	0	0	0
**14**	2	0	1	1	0	0	0	1	0	0	0	0
**15**	3	0	0	0	0	0	1	0	0	0	0	0
**16**	3	0	0	0	0	0	1	0	0	0	0	0
**17**	3	0	0	0	0	0	1	0	0	0	0	0
**18**	1	0	0	1	0	0	1	1	0	0	0	0
**19**	1	0	1	0	0	0	1	2	0	0	0	0
**20**	2	0	1	0	1	0	0	1	0	0	0	0
**21**	2	0	1	0	0	0	1	1	1	0	0	0
**22**	2	0	1	0	0	0	1	1	1	0	0	0
**23**	2	0	1	0	0	0	0	1	0	0	0	0
**24**	1	0	2	0	0	0	0	1	0	0	0	0
**25**	3	0	0	0	0	0	1	0	0	0	0	0

**26**	1	0	1	0	1	0	1	1	0	0	0	0
**27**	2	0	0	0	1	0	0	0	0	1	0	0
**28**	4	0	0	0	0	0	1	0	0	0	0	0
**29**	0	0	2	0	0	0	0	3	0	0	0	0
**30**	1	0	0	0	1	0	1	0	0	0	0	0
**31**	2	0	1	0	1	0	0	1	1	0	0	0
**32**	1	0	1	0	0	0	1	1	0	0	0	0
**33**	2	0	1	0	0	0	1	1	0	0	0	0
**34**	2	0	1	0	0	0	1	1	1	0	0	0
**35**	2	0	1	0	0	0	1	1	1	0	0	0
**36**	1	0	1	0	0	0	1	2	1	0	0	0
**37**	1	0	1	0	0	0	1	2	0	0	0	0
**38**	2	0	0	1	0	0	1	0	1	0	0	0
**39**	2	0	0	1	0	0	0	0	2	0	0	0
**40**	2	0	1	0	0	0	1	1	0	0	0	0
**41**	1	0	0	0	1	0	1	1	0	0	0	0
**42**	1	0	0	0	1	0	1	2	0	0	0	0
**43**	3	0	1	0	1	0	0	1	0	0	0	0
**44**	5	0	0	0	0	0	1	0	0	0	0	0
**45**	3	0	0	0	0	0	1	0	0	0	0	0
**46**	2	0	0	1	0	0	1	0	0	0	0	0
**47**	1	0	1	0	0	0	1	2	0	0	0	0
**48**	3	0	1	0	0	0	0	1	2	0	0	0
**49**	2	0	1	0	0	0	1	1	0	0	0	0
**50**	1	0	1	0	2	0	0	1	0	0	0	0
**51**	1	0	1	0	2	0	1	1	0	0	0	0
**52**	1	0	1	0	2	0	1	1	0	0	0	0
**53**	1	0	1	0	2	0	1	1	0	0	0	0
**54**	1	0	1	0	3	0	0	1	0	0	0	0

**55**	1	0	1	0	3	0	0	1	0	0	0	0
**56**	1	0	1	0	2	0	0	1	0	0	0	0
**57**	1	1	0	0	3	0	1	0	0	0	0	0
**58**	1	1	0	0	2	0	0	0	0	0	0	0
**59**	1	1	0	0	2	0	0	1	0	0	0	0
**60**	1	0	1	0	2	0	2	1	0	0	0	0
**61**	1	0	1	0	2	0	2	1	0	0	0	0
**62**	1	0	0	0	1	0	0	0	0	0	0	0
**63**	1	0	1	0	3	0	1	1	0	0	0	0
**64**	1	0	1	0	2	0	0	1	0	0	0	0
**65**	1	1	1	0	2	0	0	1	0	0	0	0
**66**	1	1	1	0	2	0	0	1	0	0	0	0
**67**	0	0	1	0	0	0	1	2	0	0	0	0
**68**	1	0	0	0	1	0	1	0	0	0	0	0
**69**	1	0	0	0	0	0	1	0	0	0	0	0
**70**	1	0	0	0	1	0	0	0	0	0	0	0
**71**	1	0	0	0	1	0	0	0	0	0	0	0
**72**	1	0	0	0	1	0	0	0	0	0	0	0
**73**	1	0	0	0	1	0	1	0	0	0	0	0
**74**	0	0	0	0	1	0	1	1	0	0	0	0
**75**	0	0	0	0	1	0	1	2	0	0	0	0
**76**	1	0	0	0	1	0	1	1	0	0	0	0
**77**	1	0	0	0	0	0	1	1	0	0	0	0
**78**	1	0	0	0	1	0	0	1	0	0	0	0
**79**	1	0	0	0	0	0	0	1	0	0	0	0
**80**	2	0	0	0	0	0	0	1	1	0	0	0
**81**	1	0	0	0	0	0	0	1	0	0	0	0
**82**	1	0	1	0	2	0	1	1	0	0	0	0
**83**	1	0	1	0	3	0	1	0	0	0	0	0

**84**	1	0	1	0	2	0	1	1	0	0	0	0
**85**	1	0	0	0	0	0	0	2	0	0	0	0
**86**	1	0	0	0	1	0	0	1	0	0	0	0
**87**	1	0	1	0	0	0	0	1	0	0	0	0
**88**	1	0	0	0	0	0	0	2	0	0	0	0
**89**	1	0	1	0	3	0	1	0	0	0	0	0
**90**	1	0	0	0	2	0	1	0	1	0	0	0
**91**	1	0	0	0	1	0	1	2	0	0	0	0
**92**	1	0	0	0	2	0	1	0	0	0	0	0
**93**	1	0	0	0	0	0	0	2	0	0	0	0
**94**	1	0	1	0	2	0	1	0	0	0	0	0
**95**	1	0	0	0	3	0	1	0	0	0	0	0
**96**	1	0	0	0	1	0	0	2	0	0	0	0
**97**	0	0	0	0	0	0	0	3	1	0	0	0
**98**	1	0	0	0	1	0	0	1	0	0	0	0
**99**	2	0	0	0	1	0	0	0	0	0	0	0
**100**	2	0	0	0	1	0	1	0	0	0	0	0
**101**	1	0	0	0	1	0	0	1	0	0	0	0
**102**	2	0	0	0	1	0	0	0	0	0	0	0
**103**	0	0	0	0	0	0	0	2	1	0	0	0
**104**	2	0	0	0	1	0	0	0	0	0	0	0
**105**	2	0	0	0	1	0	1	0	0	0	0	0
**106**	2	0	0	0	2	0	1	0	0	0	0	0
**107**	1	0	0	0	0	0	0	1	0	0	0	0
**108**	2	0	0	0	1	0	0	0	1	0	0	0
**109**	1	0	1	0	2	0	1	0	0	0	0	0
**110**	1	0	0	0	0	0	0	2	0	0	0	0
**111**	1	0	0	0	0	0	0	1	0	0	0	0
**112**	1	0	0	0	0	0	0	1	0	0	0	0

**113**	1	0	0	0	0	0	0	1	0	0	0	0
**114**	1	0	1	0	2	0	1	0	0	0	0	0
**115**	1	0	1	0	1	0	1	1	0	0	0	0
**116**	1	0	0	0	2	0	1	1	0	0	0	0
**117**	1	0	1	0	2	0	1	0	0	0	0	0
**118**	1	0	0	0	3	0	1	0	0	0	0	0
**119**	1	0	0	0	2	0	1	0	1	0	0	0
**120**	1	0	0	0	0	0	0	2	0	0	0	0
**121**	2	0	2	0	1	0	2	2	0	0	0	0
**122**	2	2	4	0	2	0	0	0	0	0	0	0
**123**	2	2	2	0	4	0	0	2	0	0	0	0
**124**	2	2	2	0	4	0	1	2	0	0	0	0
**125**	2	2	2	0	4	0	1	2	0	0	0	0
**126**	5	1	2	0	2	0	1	2	0	0	0	0
**127**	2	2	2	0	5	0	0	2	0	0	0	0
**128**	2	2	2	0	5	0	0	2	0	0	0	0
**129**	2	2	2	0	4	0	0	2	0	0	0	0
**130**	2	2	2	0	4	0	0	2	0	0	0	0
**131**	4	0	1	0	1	0	1	1	0	0	1	1
**132**	4	0	1	0	1	0	1	1	0	0	1	1
**133**	2	2	3	0	2	1	0	0	0	0	0	0
**134**	1	1	0	0	0	0	2	5	0	0	0	0
**135**	2	2	2	0	4	0	0	0	0	0	0	0
**136**	2	2	2	0	4	0	0	0	0	0	0	0
**137**	2	2	3	0	3	0	0	0	0	0	0	0
**138**	2	2	2	0	4	0	0	0	0	0	0	0
**139**	2	2	3	0	3	0	0	0	0	0	0	0
**140**	2	2	2	0	4	0	0	2	0	0	0	0
**141**	2	2	4	0	2	0	0	0	0	0	0	0

**142**	2	2	2	0	3	0	0	1	0	0	0	0
**143**	2	2	2	0	3	0	0	1	0	0	0	0
**144**	2	2	2	0	3	0	0	1	0	0	0	0
**145**	2	2	2	0	3	0	0	1	0	0	0	0
**146**	2	2	2	0	3	0	0	1	0	0	0	0
**147**	2	2	2	0	3	0	0	1	0	0	0	0
**148**	2	2	2	0	3	0	0	1	0	0	0	0
**149**	2	0	2	0	9	0	0	0	0	0	0	0
**150**	4	0	1	0	1	0	1	1	0	0	1	1
**151**	4	0	1	0	1	0	1	1	0	0	1	1
**152**	4	0	1	1	0	1	1	1	0	0	0	0
**153**	3	0	1	1	0	1	2	1	0	0	0	0
**154**	2	2	2	0	4	0	0	2	0	0	0	0
**155**	1	0	1	0	0	1	0	5	0	0	0	0
**156**	4	1	0	1	1	0	1	1	0	1	0	1
**157**	2	2	2	0	4	0	0	2	0	0	0	0
**158**	2	2	2	0	4	0	0	2	0	0	0	0
**159**	2	0	2	0	0	0	0	0	0	0	1	1
**160**	1	0	3	0	1	0	0	0	0	0	1	1
**161**	1	0	2	0	0	0	0	2	0	0	1	1
**162**	2	2	3	0	2	1	0	2	0	0	0	0
**163**	2	2	3	0	2	1	0	2	0	0	0	0
**164**	2	2	3	0	2	1	0	2	0	0	0	0
**165**	4	0	0	1	1	0	2	1	0	0	1	0
**166**	2	0	2	0	2	0	1	1	0	0	0	0
**167**	2	0	1	1	2	0	1	0	0	0	0	0
**168**	2	0	2	0	1	0	2	2	0	0	0	0
**169**	2	0	0	0	2	0	0	1	0	0	0	0
